# Supplementary material for: Longitudinal high-dimensional analysis identifies immune features associating with response to anti-PD-1 immunotherapy
Source: Nat Commun. 2023 Aug 22;14:5115. doi: 10.1038/s41467-023-40631-0 (PMC10444872; doi:10.1038/s41467-023-40631-0)
Supplement: Supplementary file 1 — Supplementary Information [file 41467_2023_40631_MOESM1_ESM.pdf]

Supplementary information

Longitudinal high-dimensional analysis identifies immune features associating with response to anti-PD-1 immunotherapy

Elaine Lai-Han Leung, Run-Ze Li, Xing-Xing Fan, Lily Yan Wang, Yan Wang, Zebo Jiang, Jumin Huang, Hu-Dan Pan, Yue Fan, Hongmei Xu, Feng Wang, Haopeng Rui, Piu Wong, Hermi Sumatoh, Michael Fehlings, Alessandra Nardin, Paul Gavine, Longen Zhou, Yabing Cao and Liang Liu

Supplementary data

&

Supplementary methods

Supplementary data

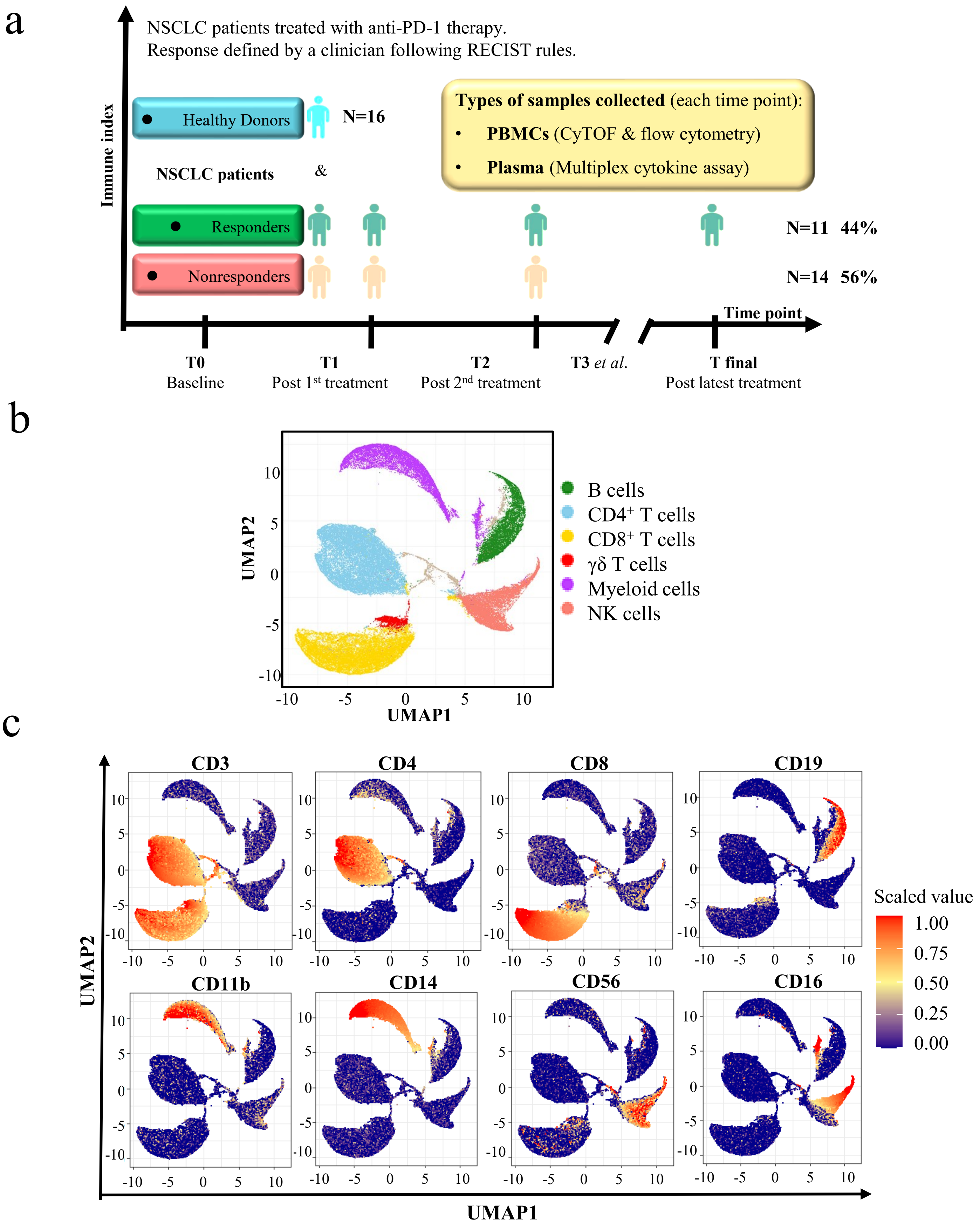

Supplementary figure 1. High-dimensional single-cell analysis of PBMCs from NSCLC patients identifies immunophenotypes. a. Illustration of the study design. The analysis was performed on PBMCs from healthy donors (n=20) and NSCLC patients (total patient number=25, responder number=11, nonresponder number=14). b. Manual clustering for major immune cell types. Cells were colored according to the cluster they were assigned to using the FlowSOM algorithm and manual annotation. c. Bioinformatic analysis of phenotypic profiling by UMAP dimension reduction and clustering algorithms (colored by sample features).

Supplementary data

CT scan

Before immunotherapy

After immunotherapy

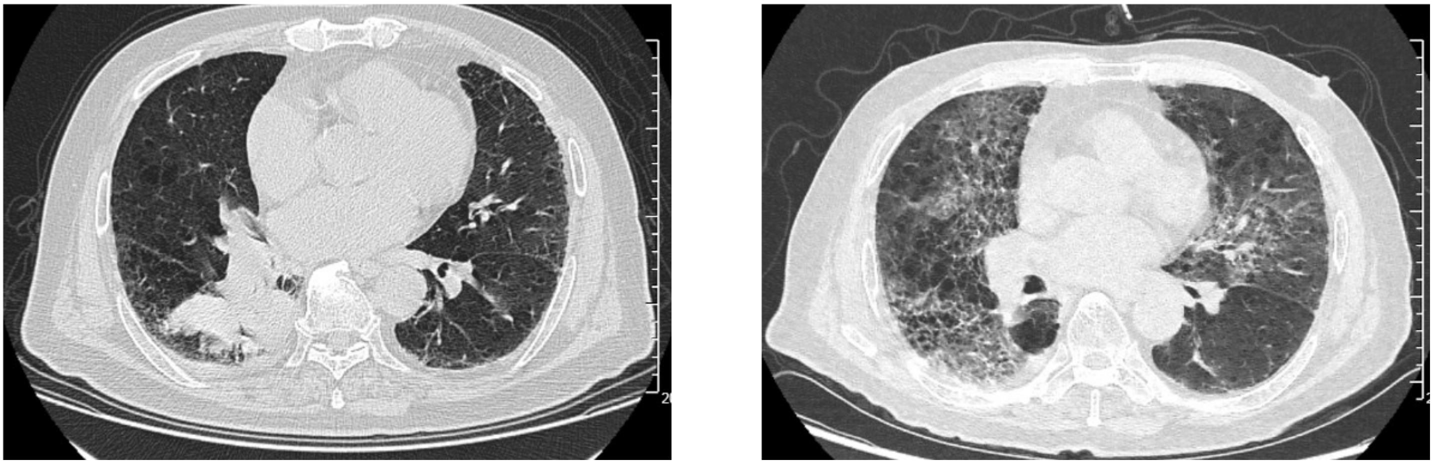

Supplementary figure 2. Representative patient CT scans. CT scan comparison for an 83-year-old patient with lung cancer before and after immunotherapy. Immune-related pneumonitis was also identified. Below, representative IHC images.

Supplementary data

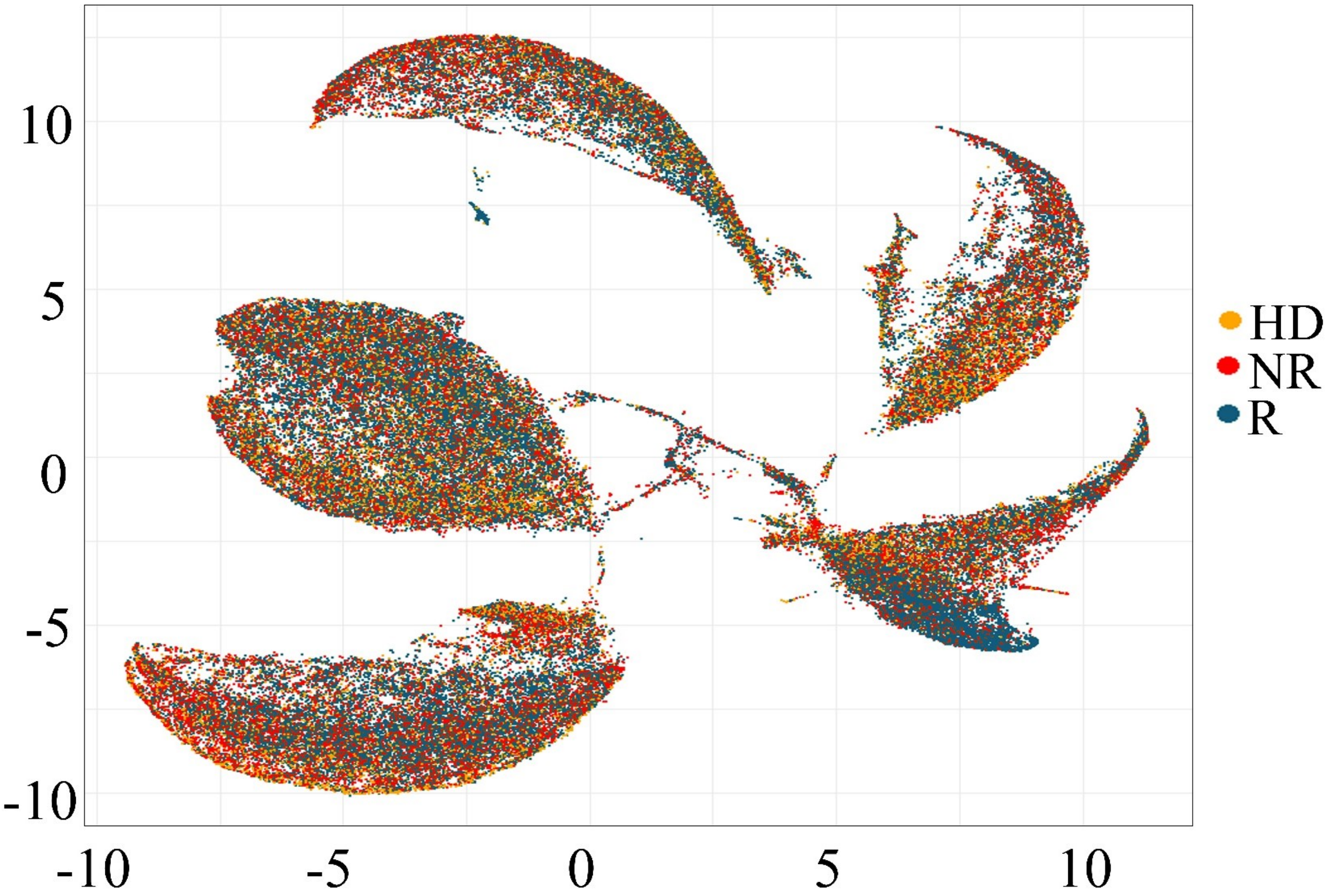

Supplementary figure 3. Exemplified UMAP visualization for healthy donors, responders and nonresponders. HD, healthy donors. NR, nonresponders. R, responders.

Supplementary data

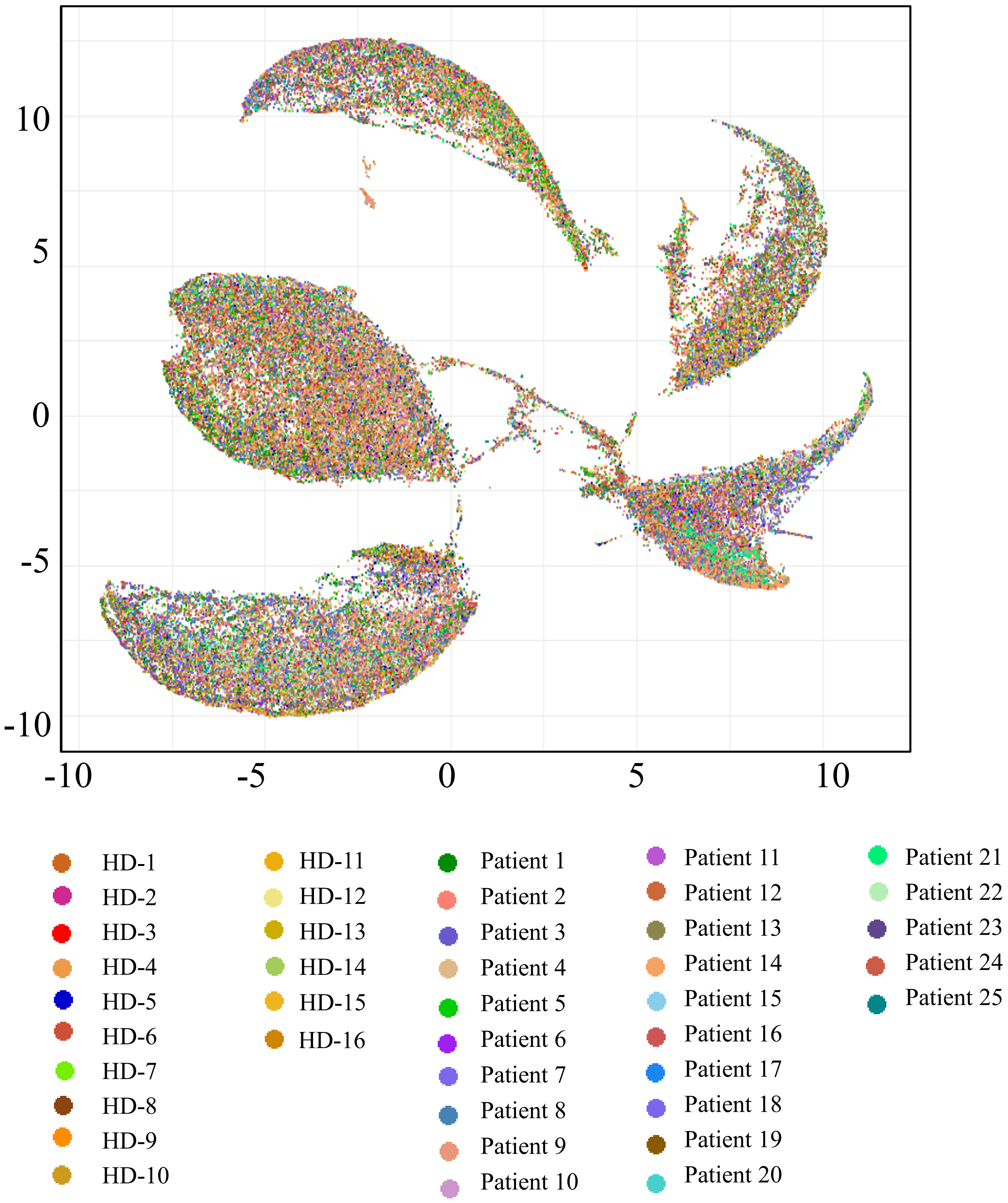

Supplementary figure 4. Exemplified UMAP visualization for different patients.

Supplementary data

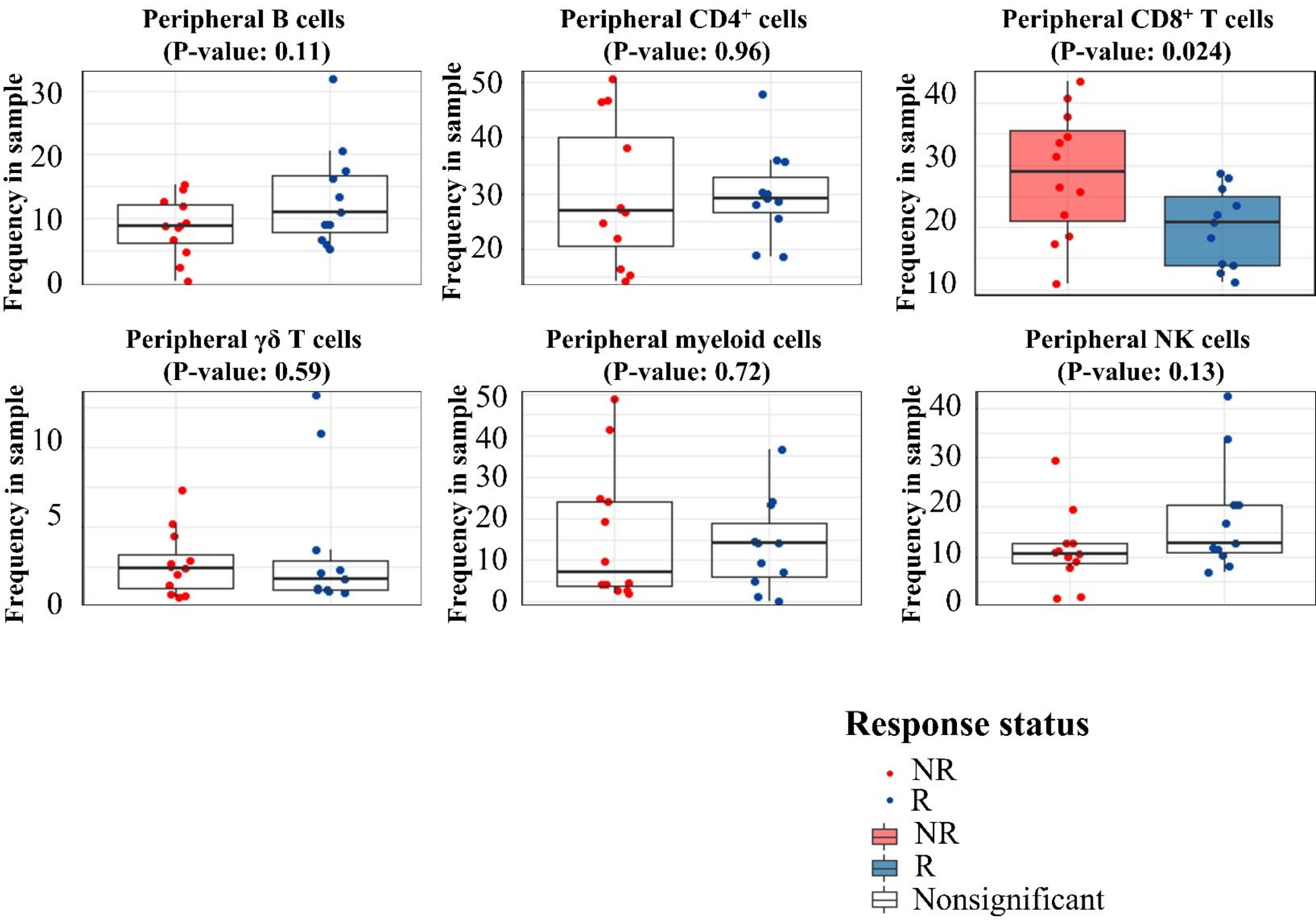

Supplementary figure 5. Overview of the main types of immune cells in responders (n=11) and nonresponders (n=14) evaluated by a physician after receipt of anti-PD-1 immunotherapy. The box plots represent the interquartile range (IQR), with the horizontal line indicating the median. The whiskers extend to the farthest data point within a maximum of 1.5 × IQR. All *p-values* were calculated using two-sided t-tests and were corrected for multiple comparisons using the Benjamini-Hochberg adjustment.

Supplementary data

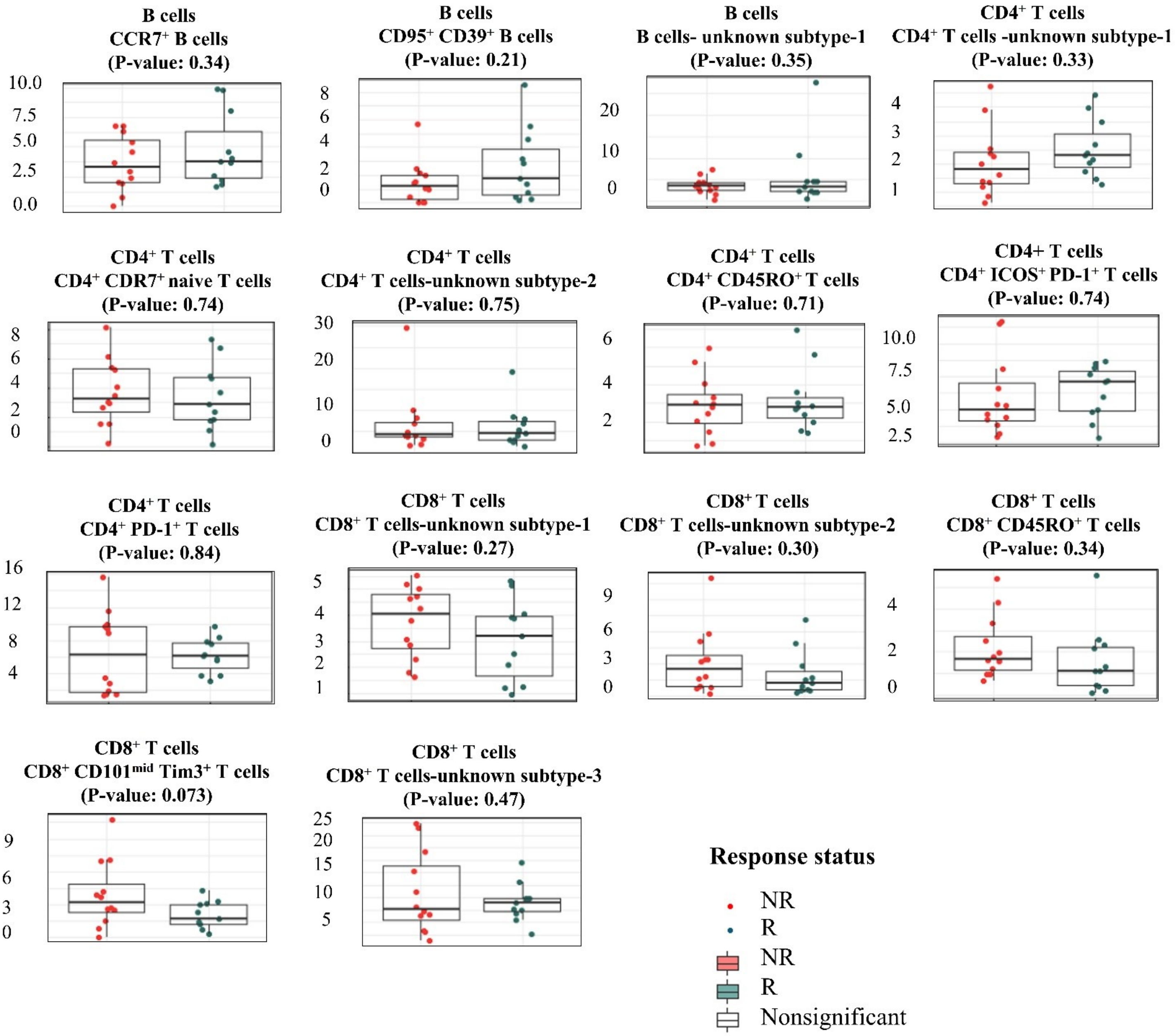

Supplementary figure 6-1. Overview of subsets of immune cells in responders (n=11) and non-responders (n=14). The box plots represent the interquartile range (IQR), with the horizontal line indicating the median. The whiskers extend to the farthest data point within a maximum of 1.5 × IQR. All *p-values* were calculated using two-sided t-tests comparing the R to the NR and were corrected for multiple comparisons using the Benjamini-Hochberg adjustment. For each unknown subset, an unique ID 1,2,3,4 were added for differentiation, which were consistent to all subsequent data.

Supplementary data

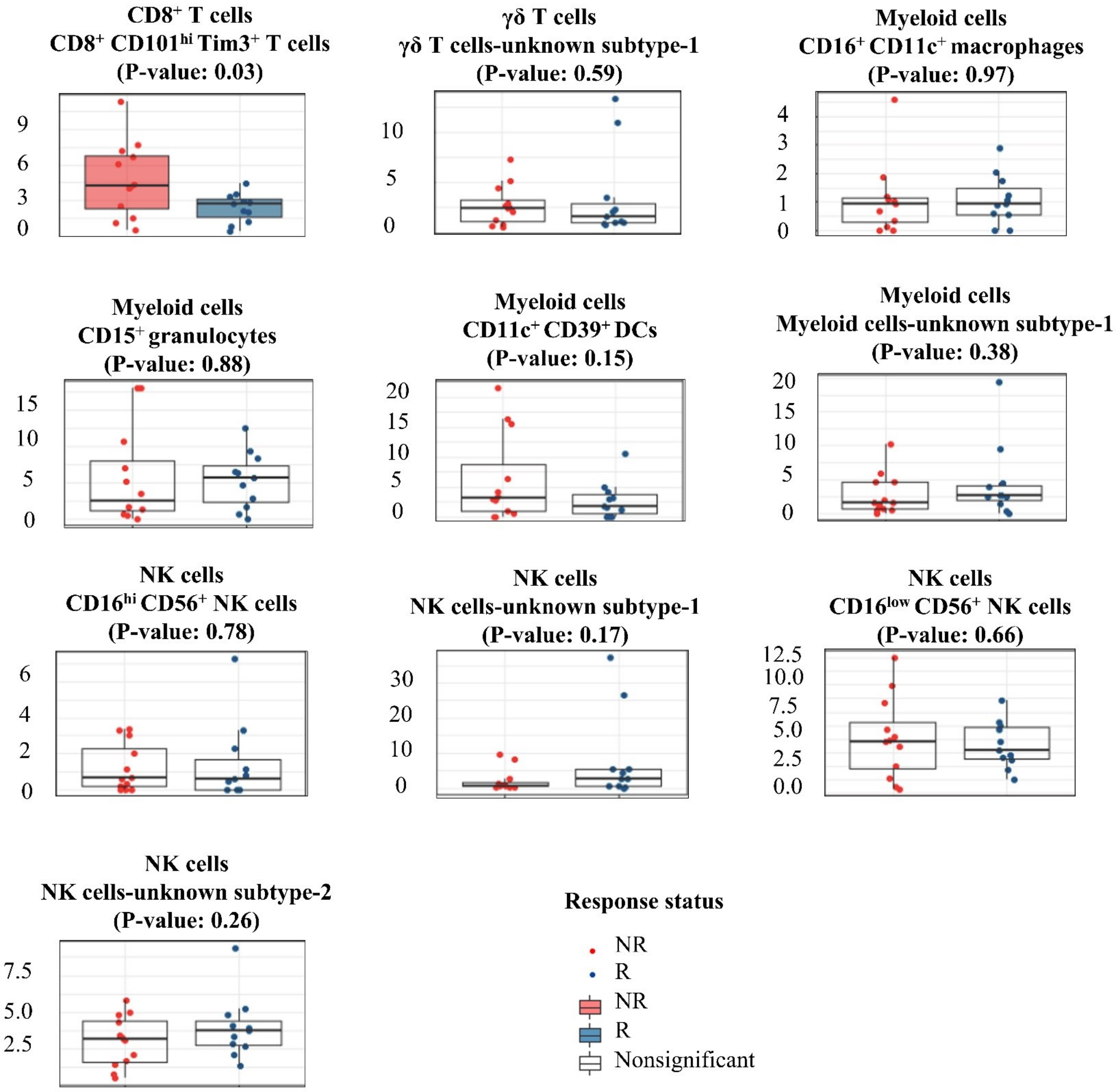

Supplementary figure 6-2. Overview of subsets of immune cells in responders (n=11) and non-responders (n=14). The box plots represent the interquartile range (IQR), with the horizontal line indicating the median. The whiskers extend to the farthest data point within a maximum of 1.5 × IQR. All *p-values* were calculated using two-sided t-tests comparing the R to the NR and were corrected for multiple comparisons using the Benjamini-Hochberg adjustment.

Supplementary data

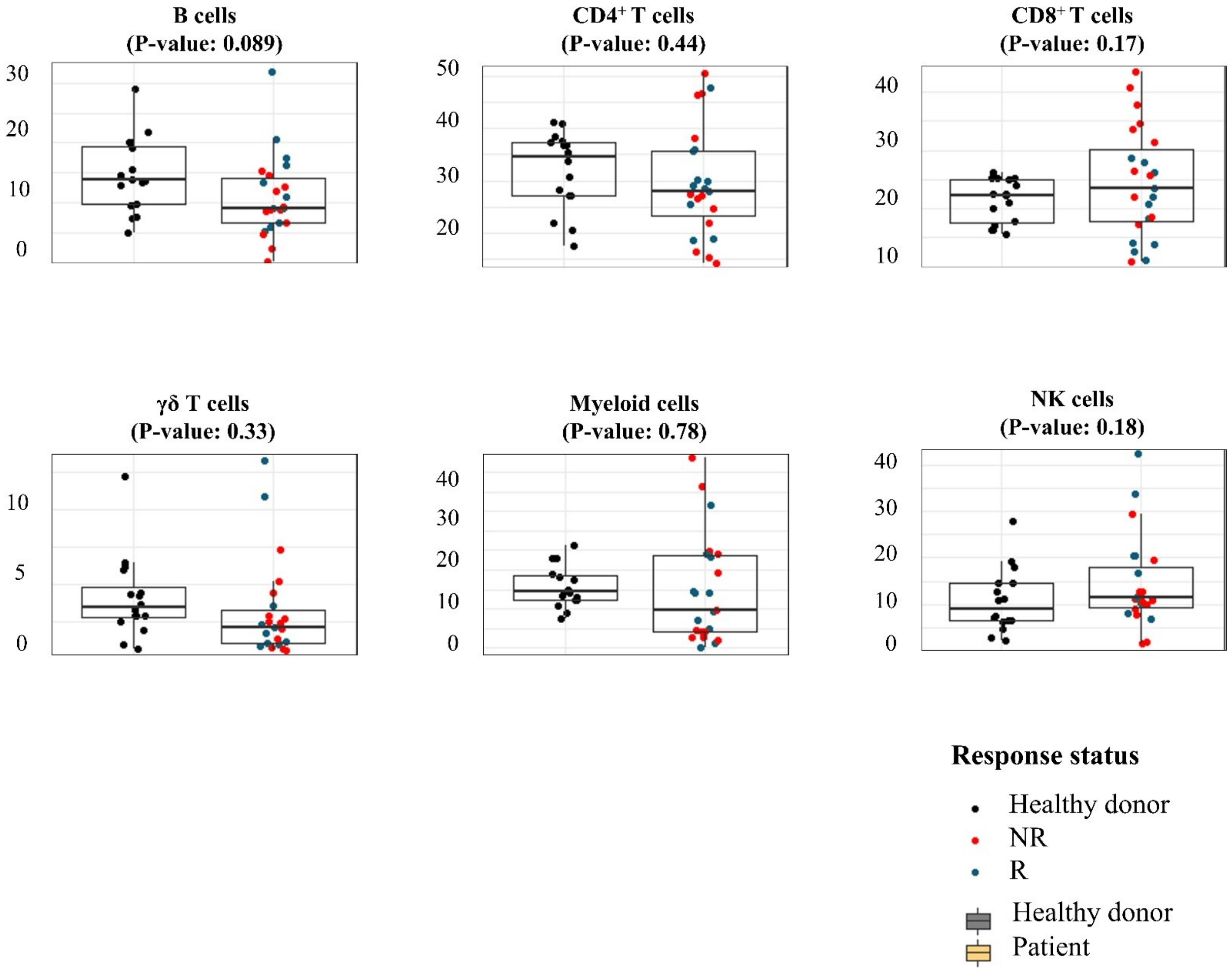

Supplementary figure 7-1. The comparison of patients (n=25) and healthy donors (n=16) (at baseline) by Major type and subsets of immune cells. The box plots represent the interquartile range (IQR), with the horizontal line indicating the median. The whiskers extend to the farthest data point within a maximum of 1.5 × IQR. All *p-values* were calculated using two-sided t-tests comparing the patients to the HDs and were corrected for multiple comparisons using the Benjamini-Hochberg adjustment.

Supplementary data

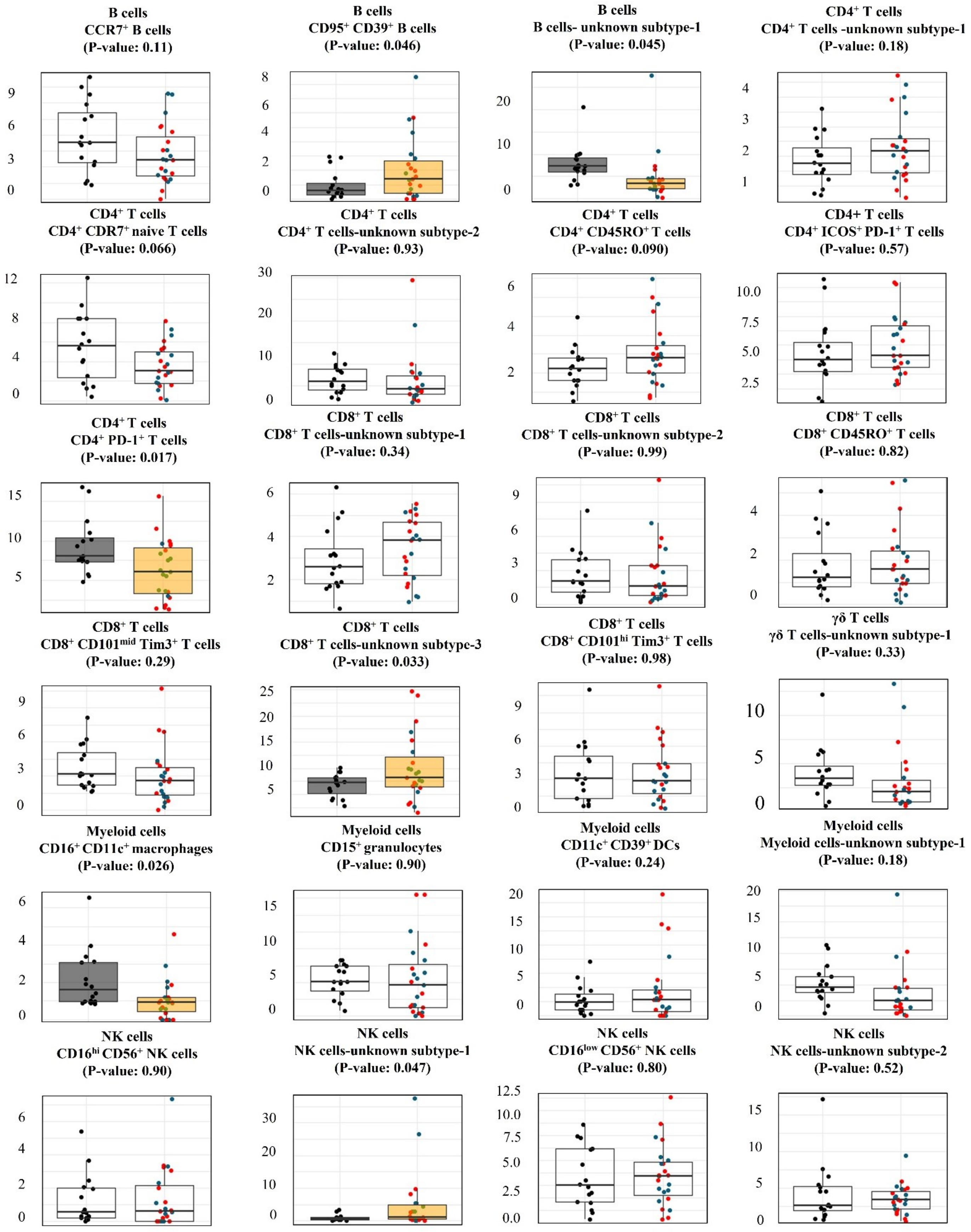

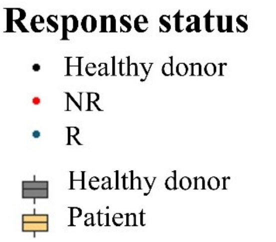

Supplementary figure 7-2. The comparison of patients (n=25) and healthy donors (n=16) (at baseline) by Major type and subsets of immune cells with a p-value < 0.05. The box plots represent the interquartile range (IQR), with the horizontal line indicating the median. The whiskers extend to the farthest data point within a maximum of 1.5 × IQR. All *p-values* were calculated using two-sided t-tests comparing the patients to the HDs and were corrected for multiple comparisons using the Benjamini-Hochberg adjustment.

Supplementary data

Normalized expression of CD101 in major immune cell populations between R and NR

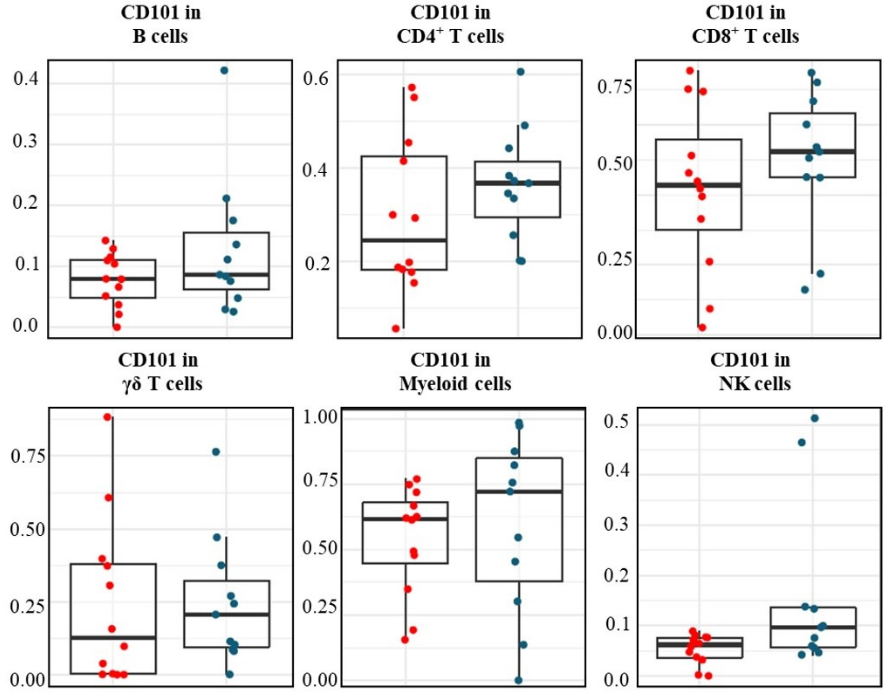

Normalized expression of TIM3 in major immune cell populations between R and NR

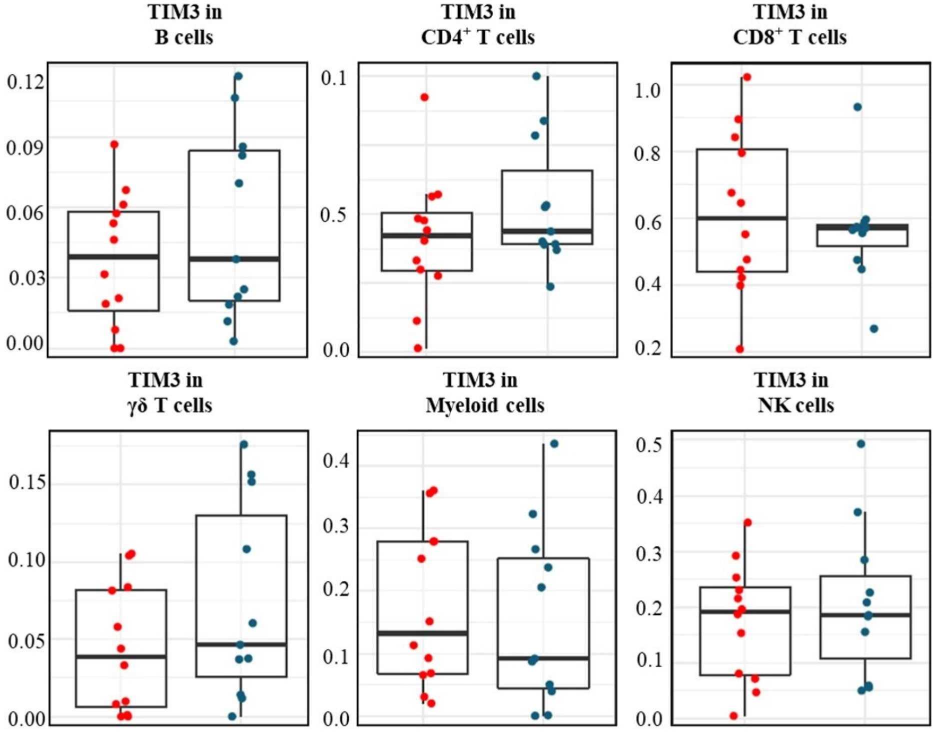

Supplementary figure 8. Relative distributions of CD101 and TIM3 expression in different major immune cell populations of responders (n=11) and non-responders (n=14). The box plots represent the interquartile range (IQR), with the horizontal line indicating the median. The whiskers extend to the farthest data point within a maximum of 1.5 × IQR.

Supplementary data

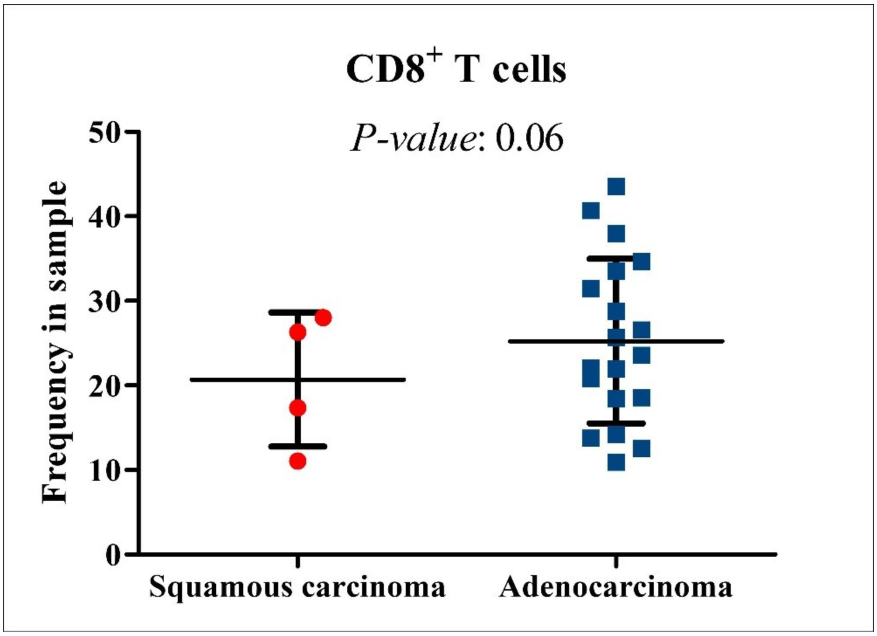

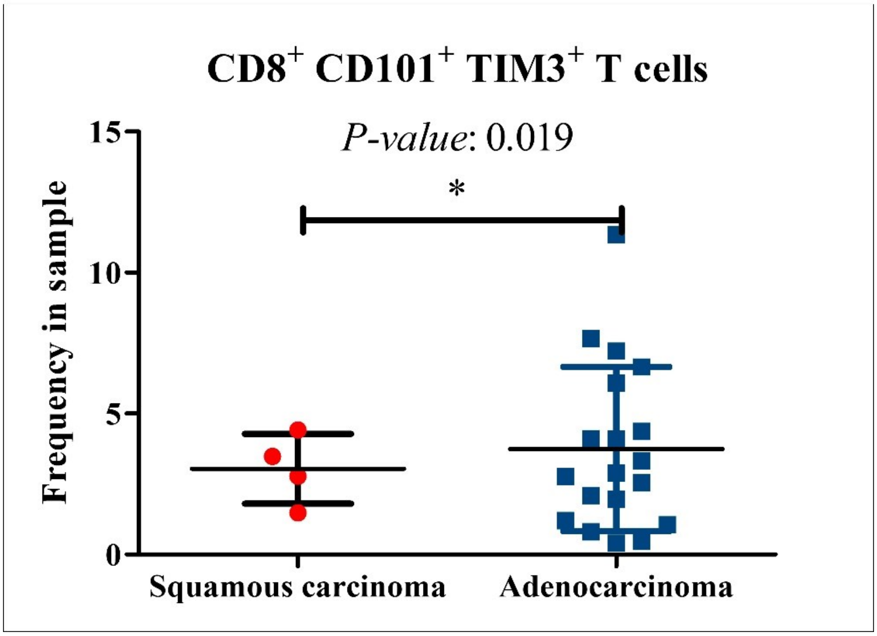

Supplementary figure 9. The comparison of frequency of $CD8^+$ T cells and CCT cells by the different histological sub-types of NSCLC in responders (n=11) and non-responders (n=14). Data are presented as mean values +/- SD. All *p-values* were calculated using two-sided t-tests comparing the patients to the HD and were corrected for multiple comparisons using the Benjamini-Hochberg adjustment

Supplementary data

Normalized expression of PD-1 in major immune cell populations between R and NR

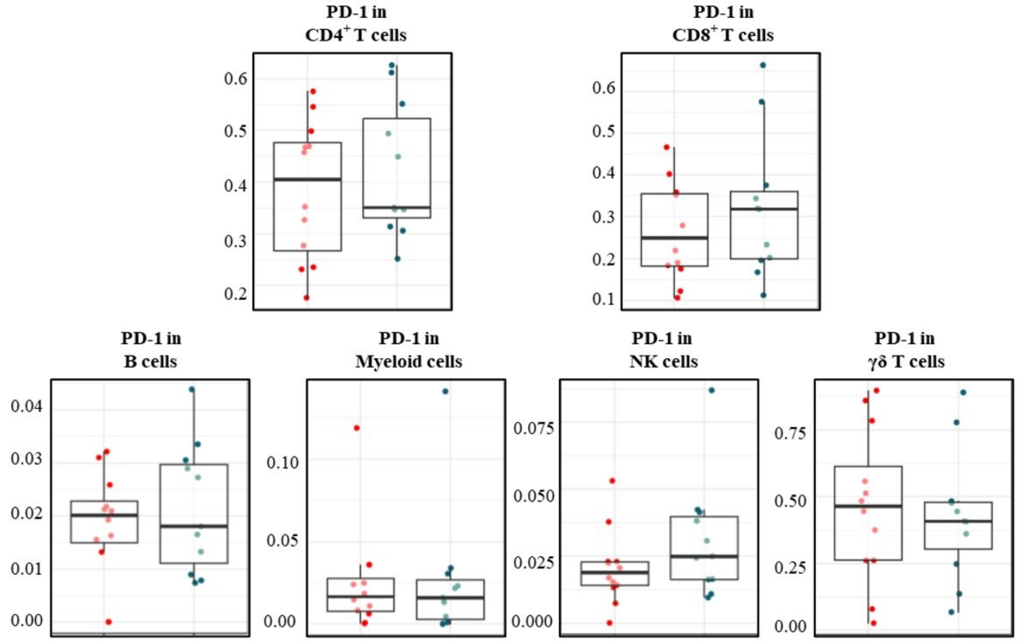

Normalized expression PD-1 in subsets

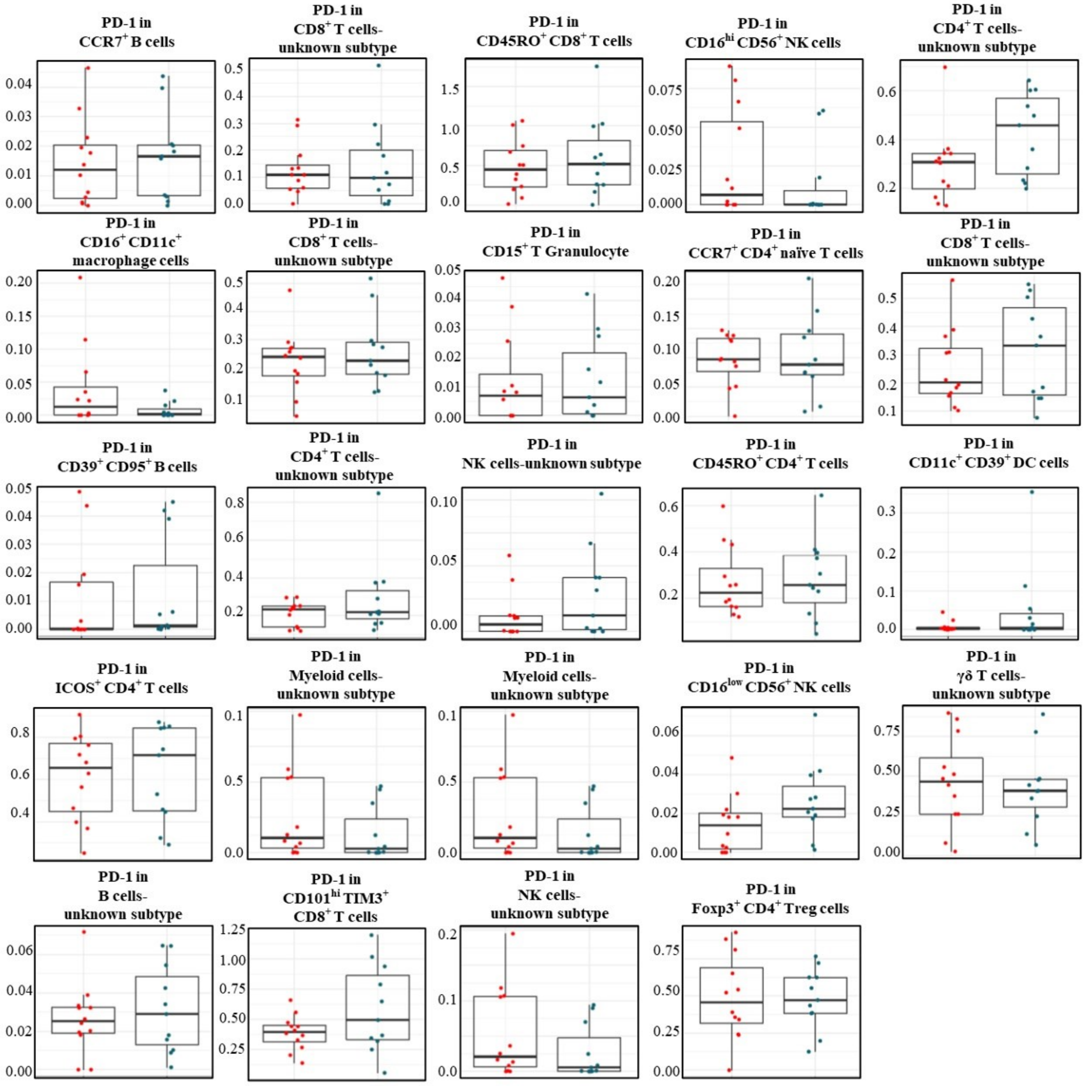

Supplementary figure 10. Relative distributions of PD1 expression in different major immune cell populations of responders (n=11) and non-responders (n=14). The box plots represent the interquartile range (IQR), with the horizontal line indicating the median. The whiskers extend to the farthest data point within a maximum of 1.5 × IQR.

Supplementary data

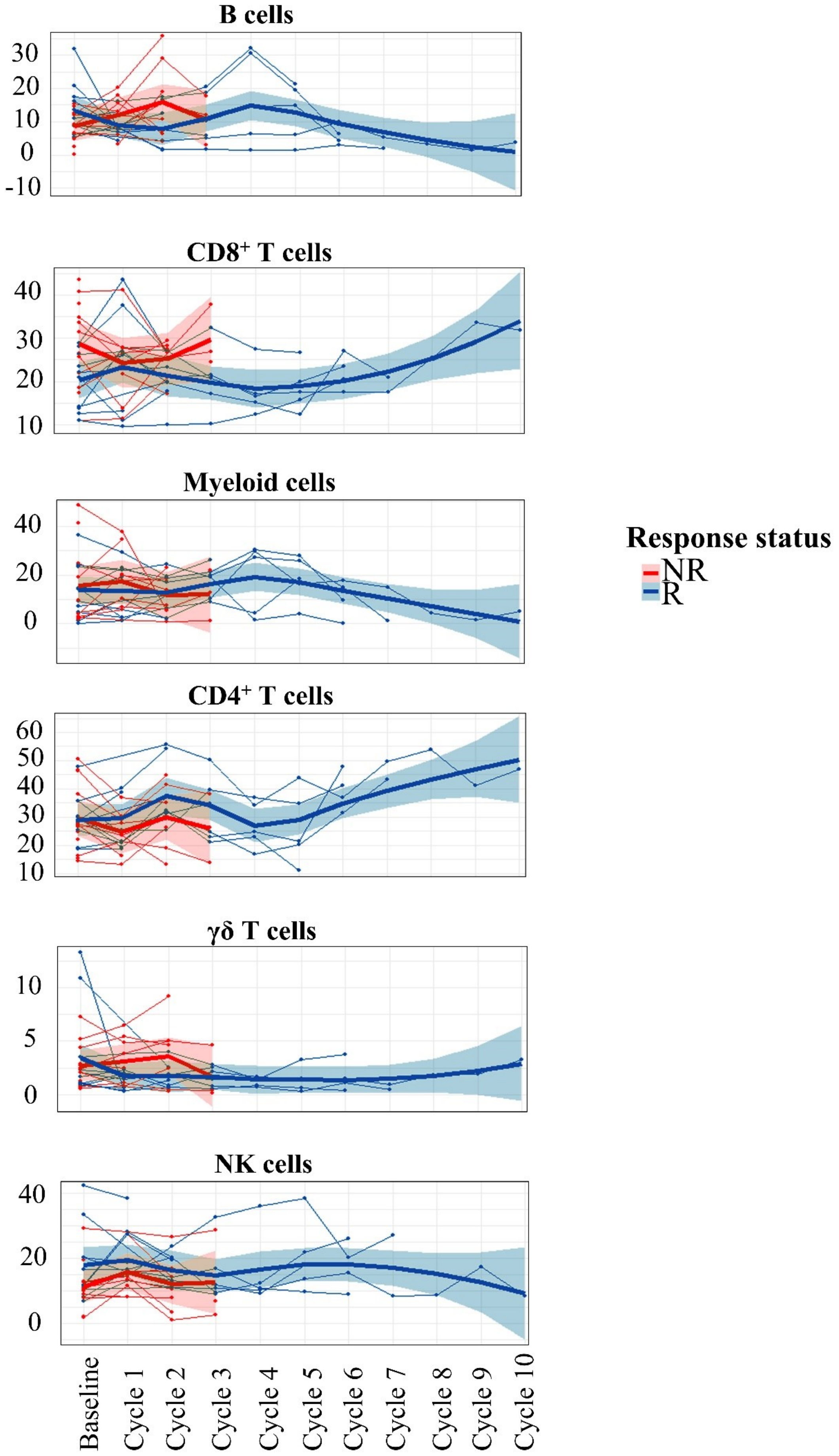

Supplementary figure 11. Longitudinal behavior of antigens in major types of immune cells compared between responders (n=11) and non-responders (n=14). Data are presented as mean values +/- SD.

Supplementary data

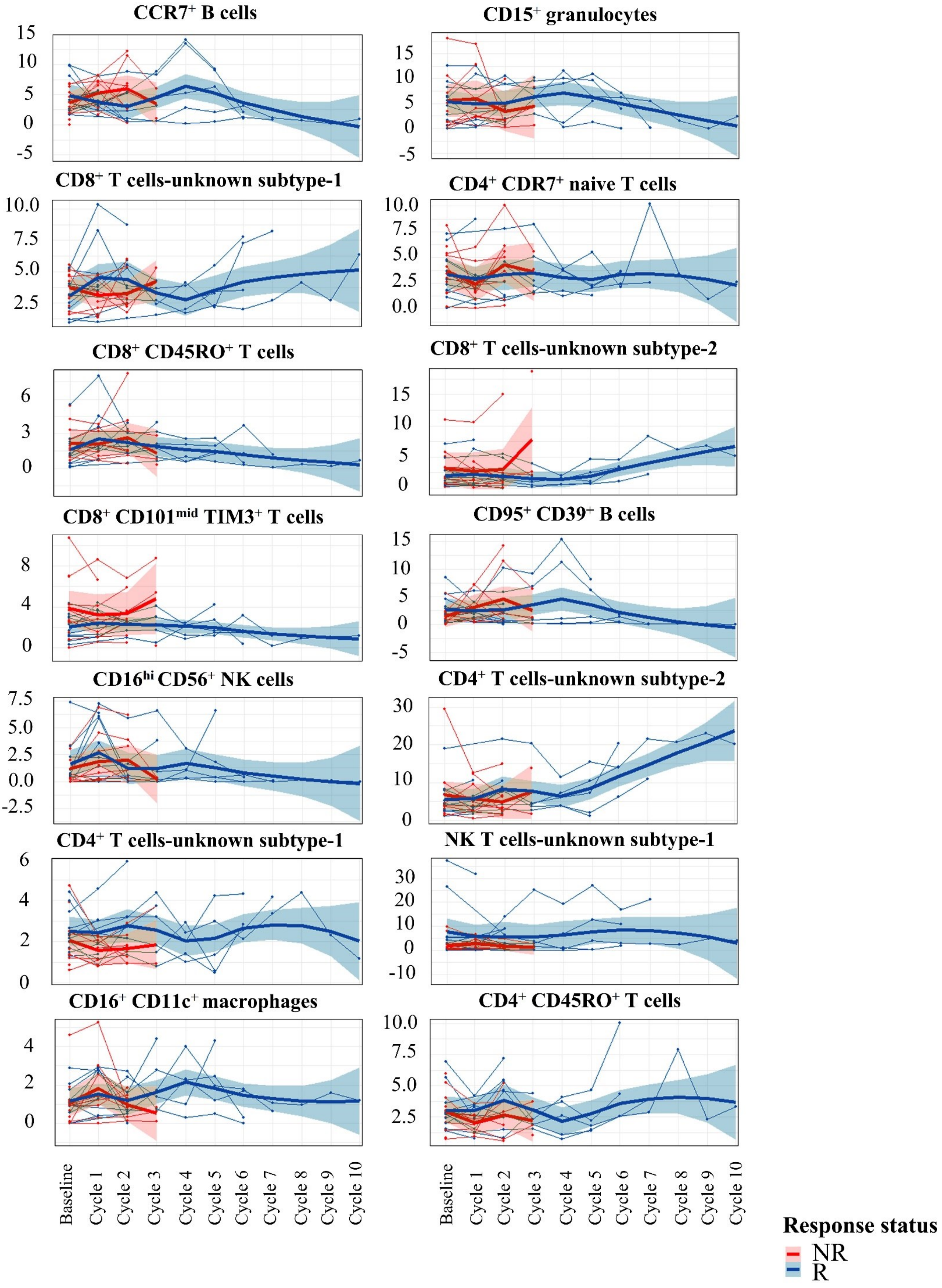

Supplementary figure 12-1. Longitudinal behavior of subtypes of immune cells compared between responders (n=11) and non-responders (n=14). Data are presented as mean values +/- SD.

Supplementary data

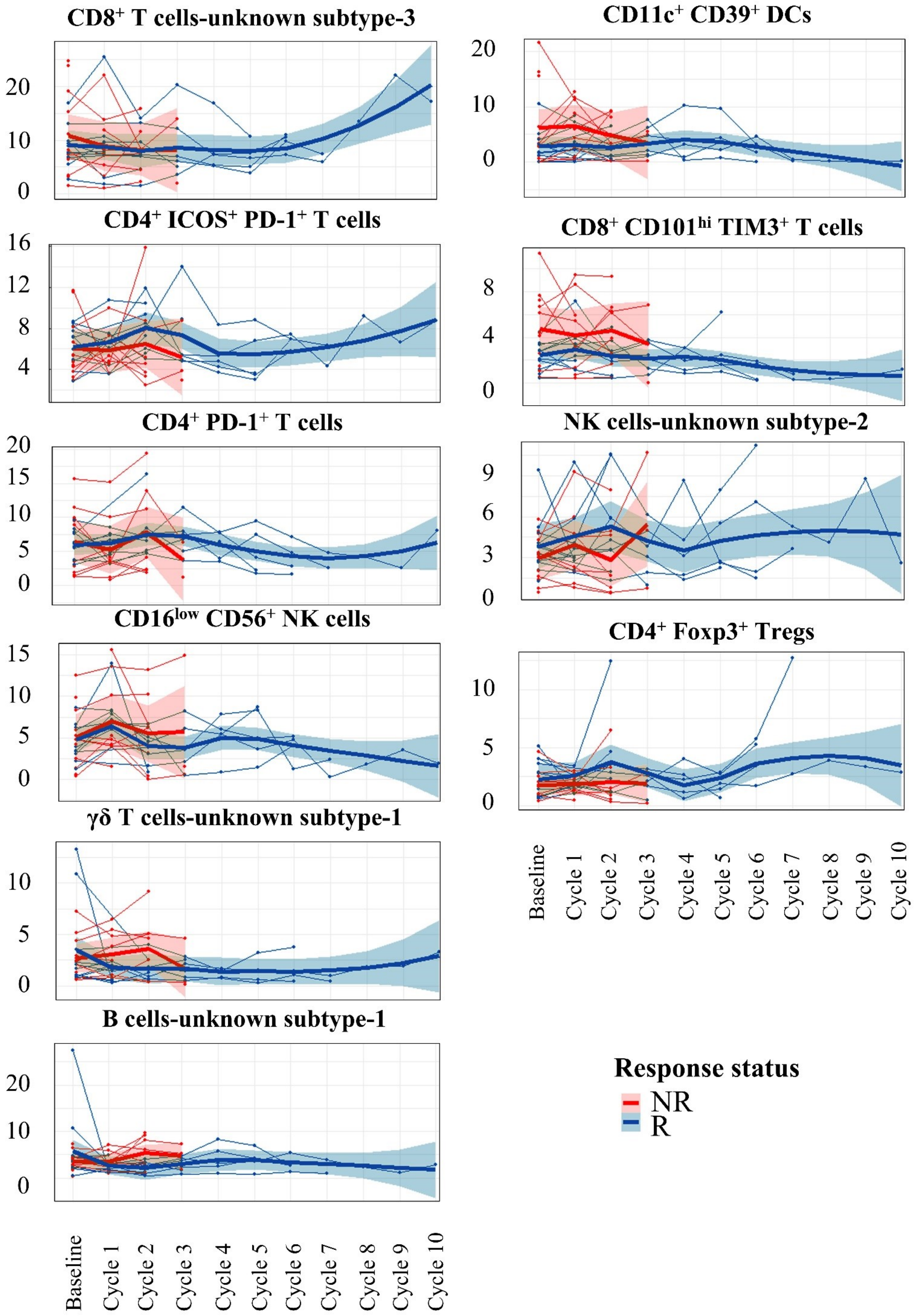

Supplementary figure 12-2. Longitudinal behavior of subtypes of immune cells compared between responders (n=11) and non-responders (n=14). Data are presented as mean values +/- SD.

Supplementary data

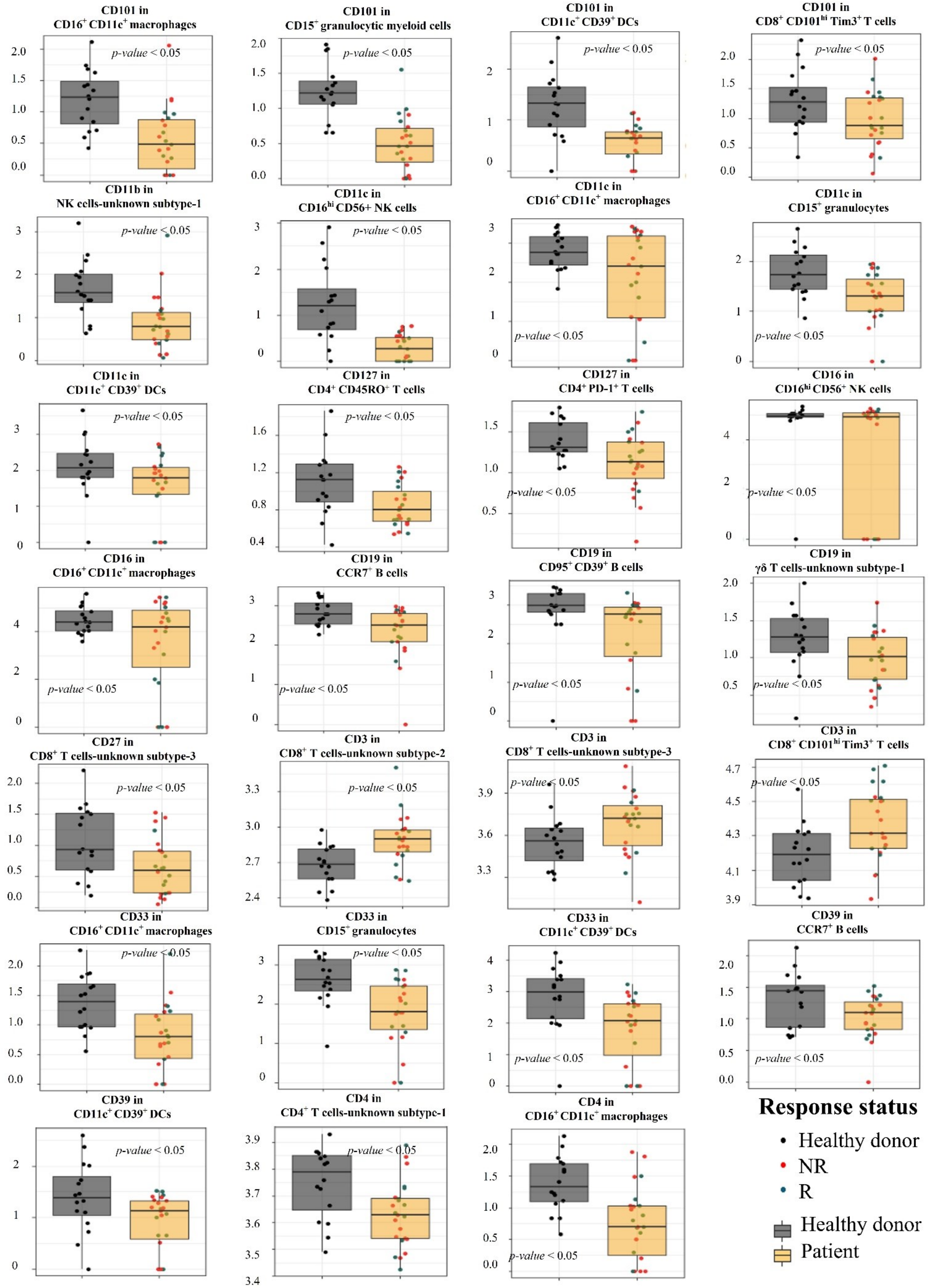

Supplementary figure 13-1. Antigens of immune cells with a *p-value* < 0.05 when comparing patients (n=25) and healthy donors (n=16) (at baseline). The box plots represent the interquartile range (IQR), with the horizontal line indicating the median. The whiskers extend to the farthest data point within a maximum of 1.5 × IQR. All *p-values* were calculated using two-sided t-tests comparing the patients to the HD and were corrected for multiple comparisons using the Benjamini-Hochberg adjustment.

Supplementary data

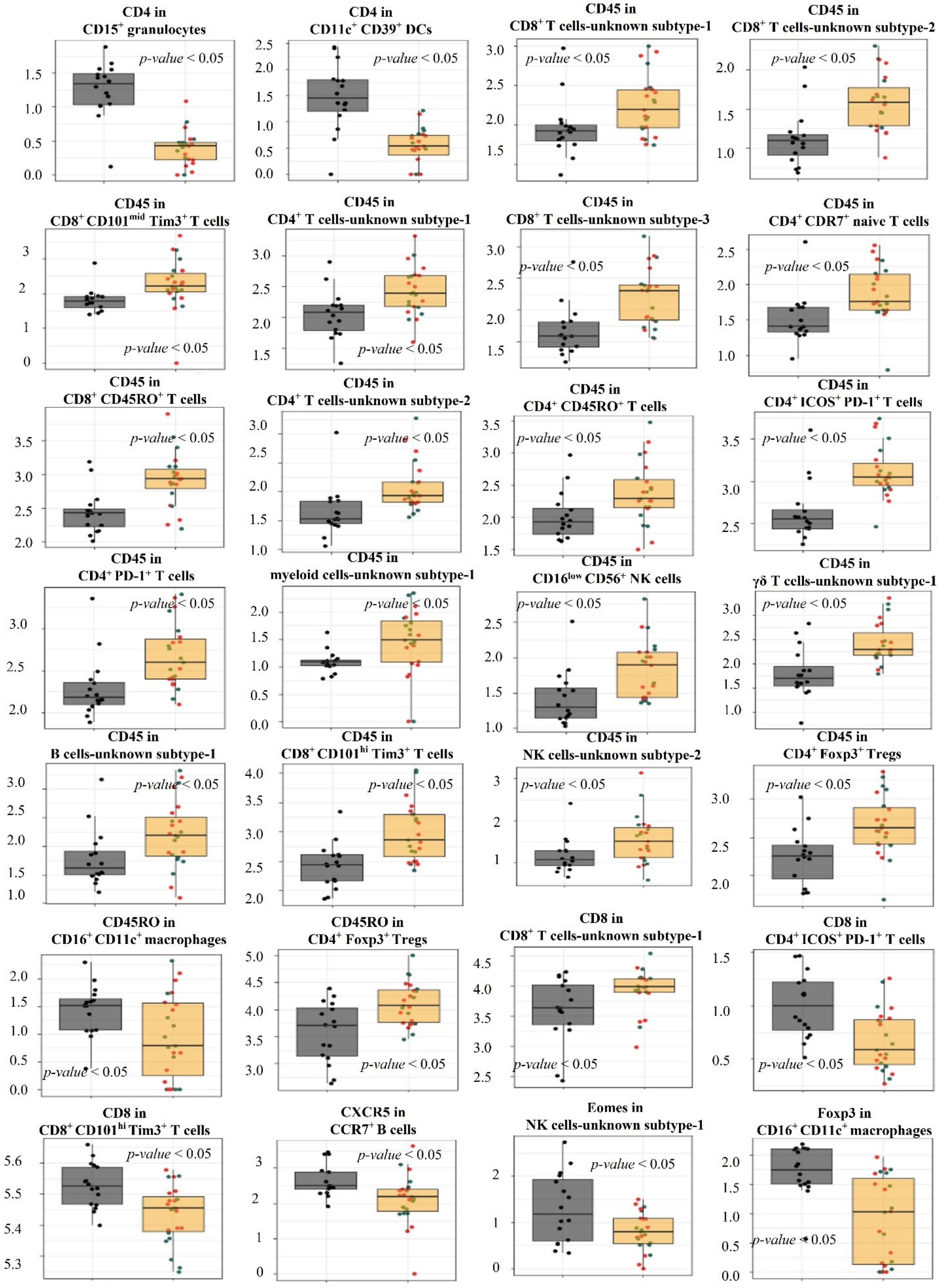

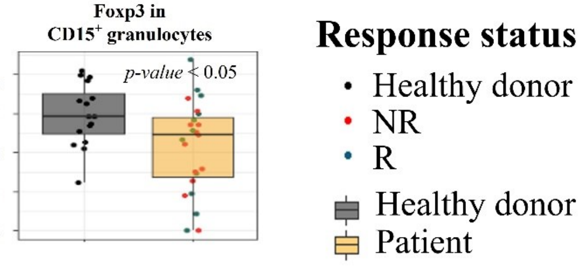

Supplementary figure 13-2. Antigens of immune cells with a *p-value* < 0.05 when comparing patients (n=25) and healthy donors (n=16) (at baseline). The box plots represent the interquartile range (IQR), with the horizontal line indicating the median. The whiskers extend to the farthest data point within a maximum of 1.5 × IQR.

Supplementary data

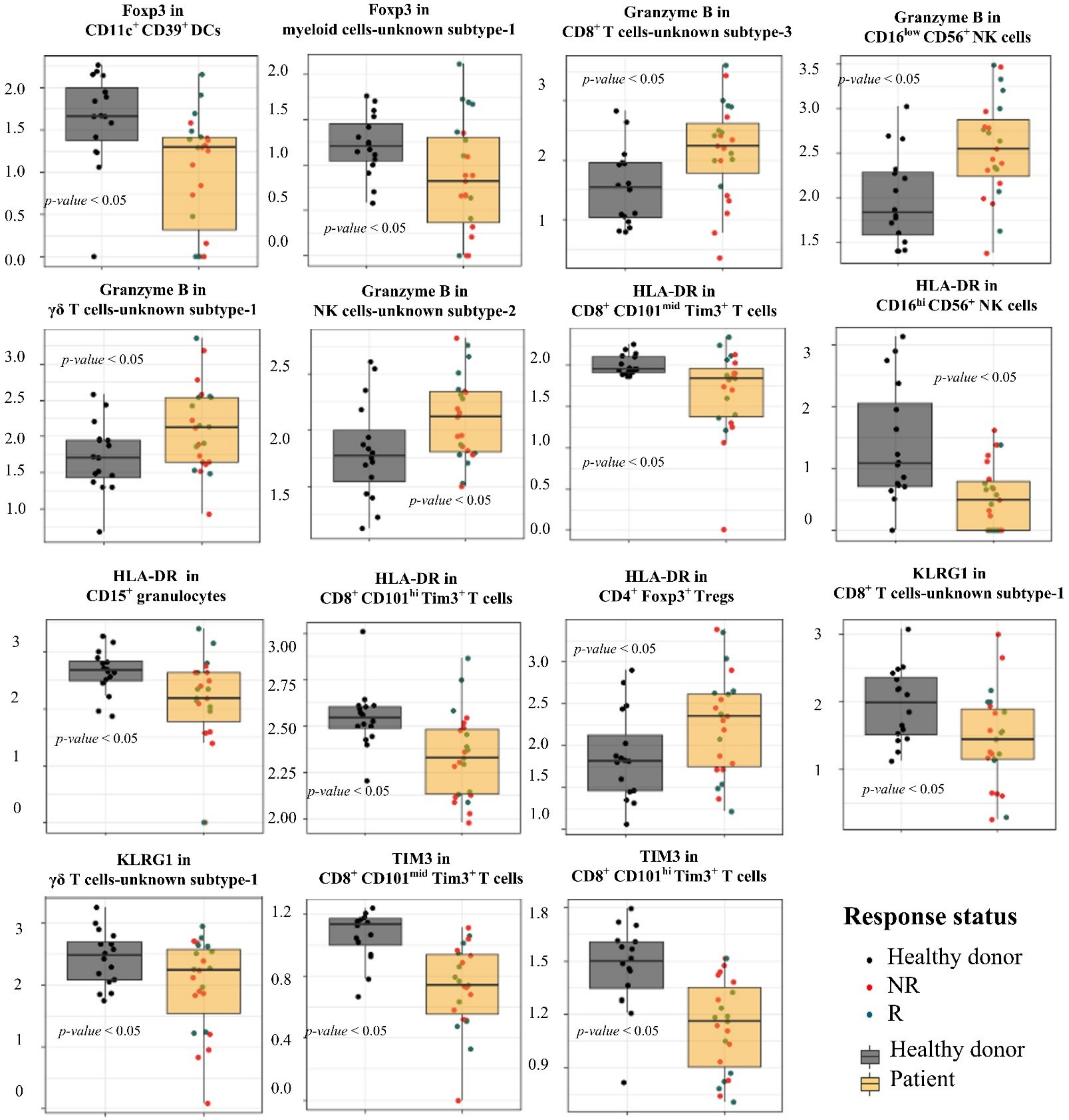

Supplementary figure 13-3. Antigens of immune cells with a p-value < 0.05 when comparing patients (n=25) and healthy donors (n=16) (at baseline). The box plots represent the interquartile range (IQR), with the horizontal line indicating the median. The whiskers extend to the farthest data point within a maximum of 1.5 × IQR. All *p-values* were calculated using two-sided t-tests comparing the patients to the HD and were corrected for multiple comparisons using the Benjamini-Hochberg adjustment.

Supplementary data

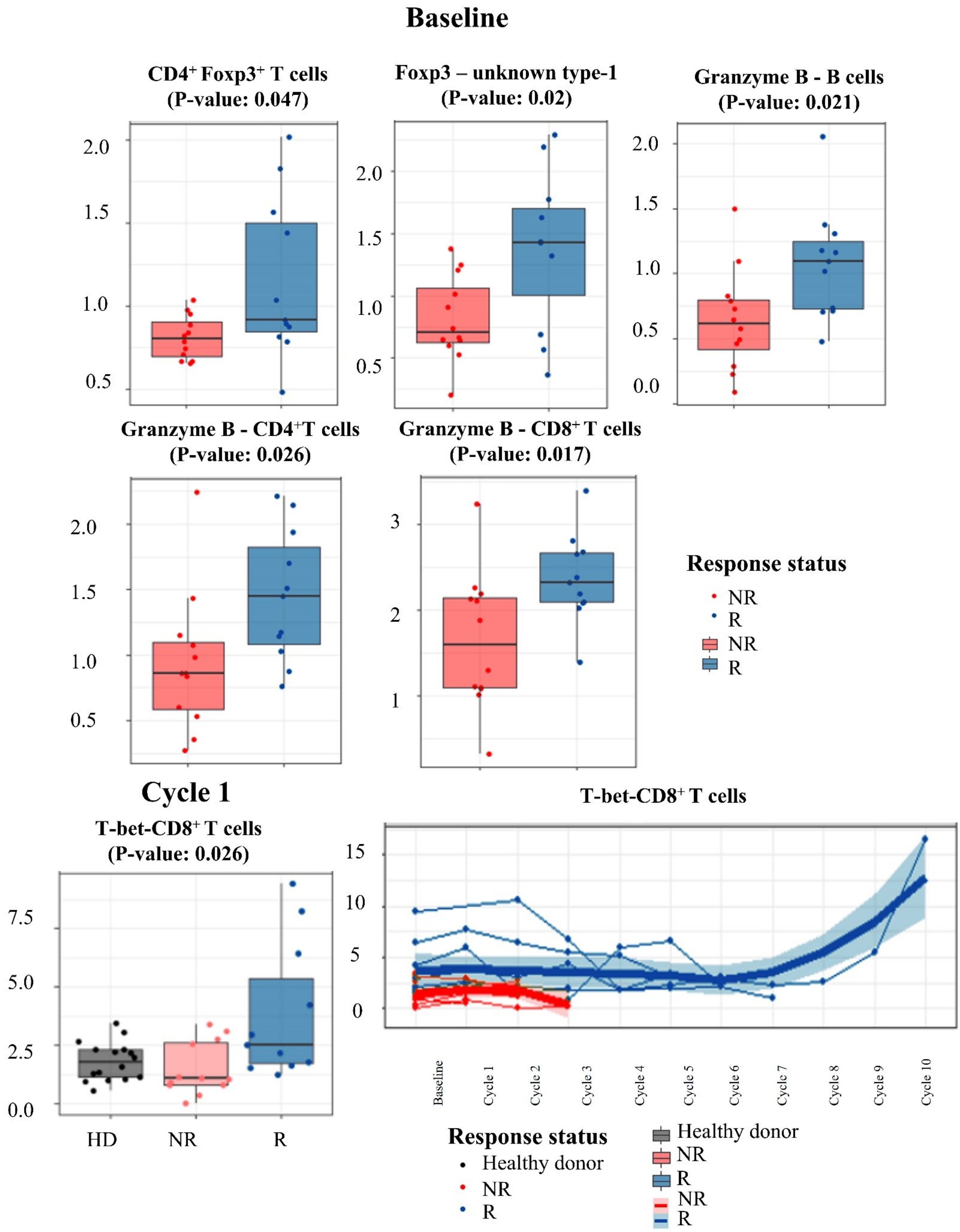

Supplementary figure 14. Antigens on major types of immune cells with a p-value < 0.05 when comparing responders (n=11) and non-responders (n=14) (at baseline and after one cycle of anti-PD1 treatment) as well as the longitudinal behavior in patients with NSCLC. The box plots represent the interquartile range (IQR), with the horizontal line indicating the median. The whiskers extend to the farthest data point within a maximum of 1.5 × IQR. All *p-values* were calculated using two-sided t-tests comparing the patients to the HD and were corrected for multiple comparisons using the Benjamini-Hochberg adjustment. Data in longitudinal analysis are presented as mean values +/- SD.

Supplementary data

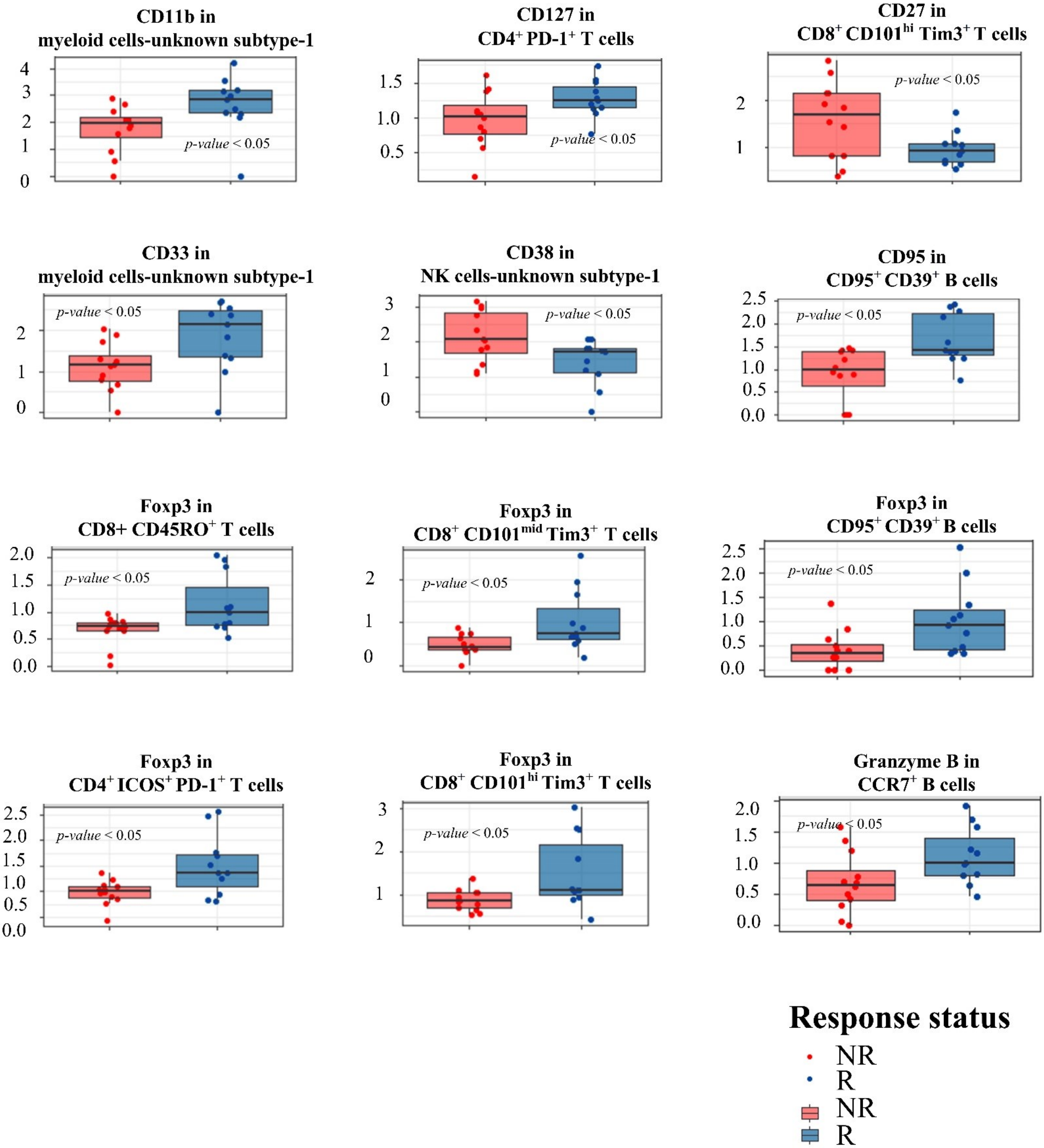

Supplementary figure 15-1. Antigens on subsets of immune cells with a *p-value* < 0.05 when comparing responders (n=11) and non-responders (n=14) (at baseline). The box plots represent the interquartile range (IQR), with the horizontal line indicating the median. The whiskers extend to the farthest data point within a maximum of 1.5 × IQR. All *p-values* were calculated using two-sided t-tests comparing the patients to the HD and were corrected for multiple comparisons using the Benjamini-Hochberg adjustment.

Supplementary data

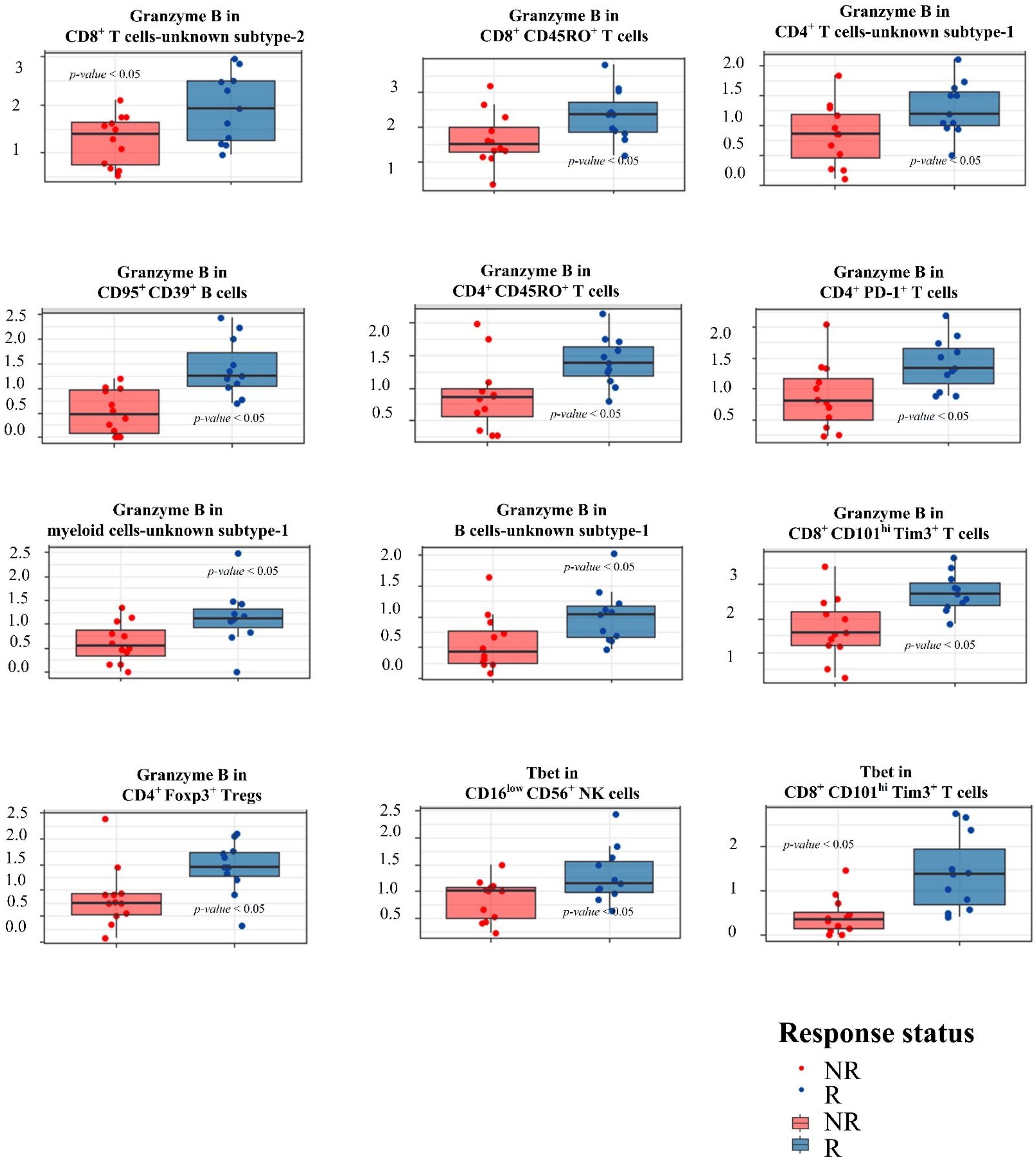

Supplementary figure 15-2. Antigens on subsets of immune cell with a *p-value* < 0.05 when comparing responders (n=11) and non-responders (n=14) (at baseline). The box plots represent the interquartile range (IQR), with the horizontal line indicating the median. The whiskers extend to the farthest data point within a maximum of 1.5 × IQR. All *p-values* were calculated using two-sided t-tests comparing the patients to the HD and were corrected for multiple comparisons using the Benjamini- [illegible]

Supplementary data

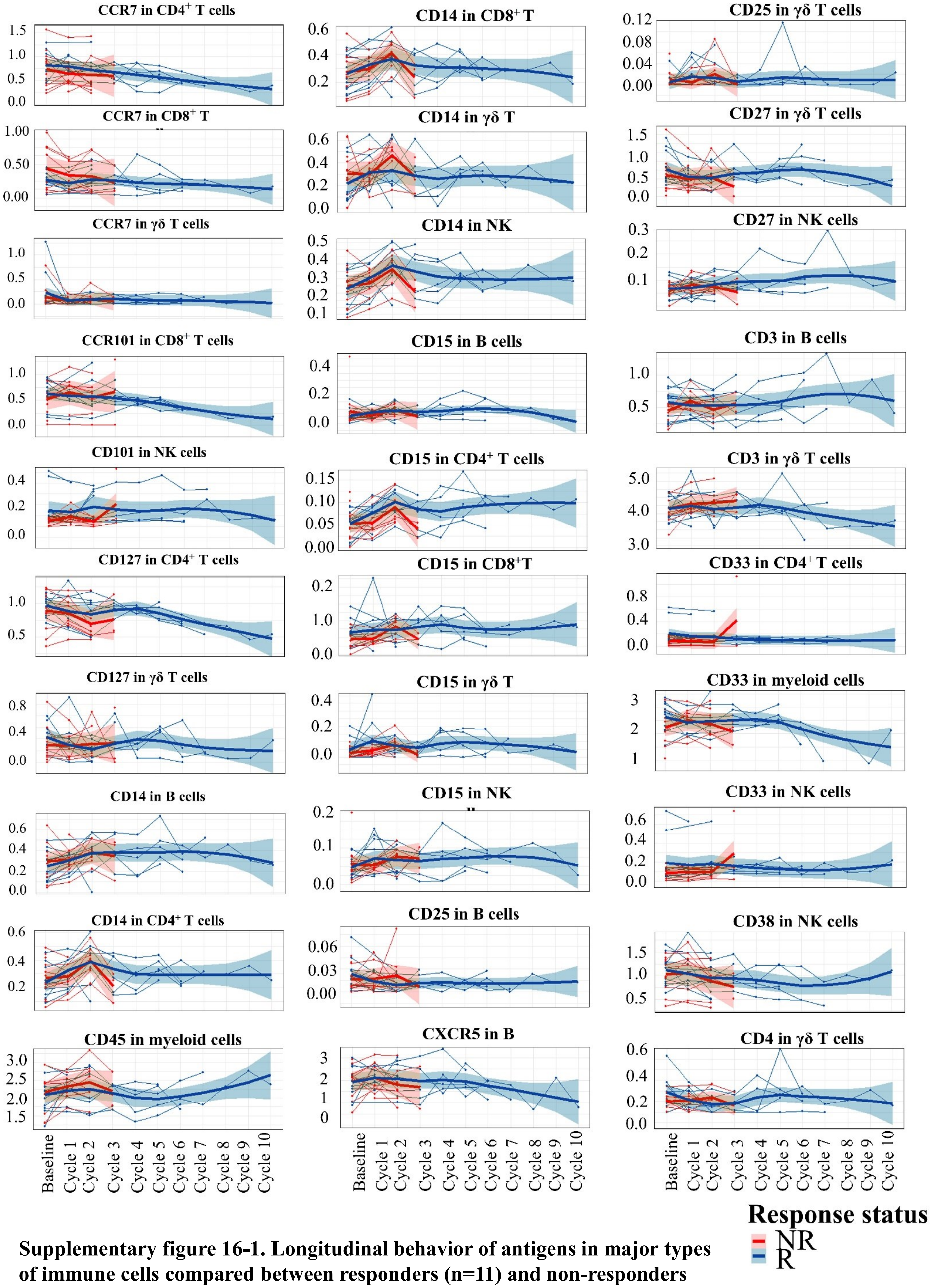

Supplementary figure 16-1. Longitudinal behavior of antigens in major types of immune cells compared between responders (n=11) and non-responders (n=14). Data in longitudinal analysis are presented as mean values +/- SD.

Supplementary data

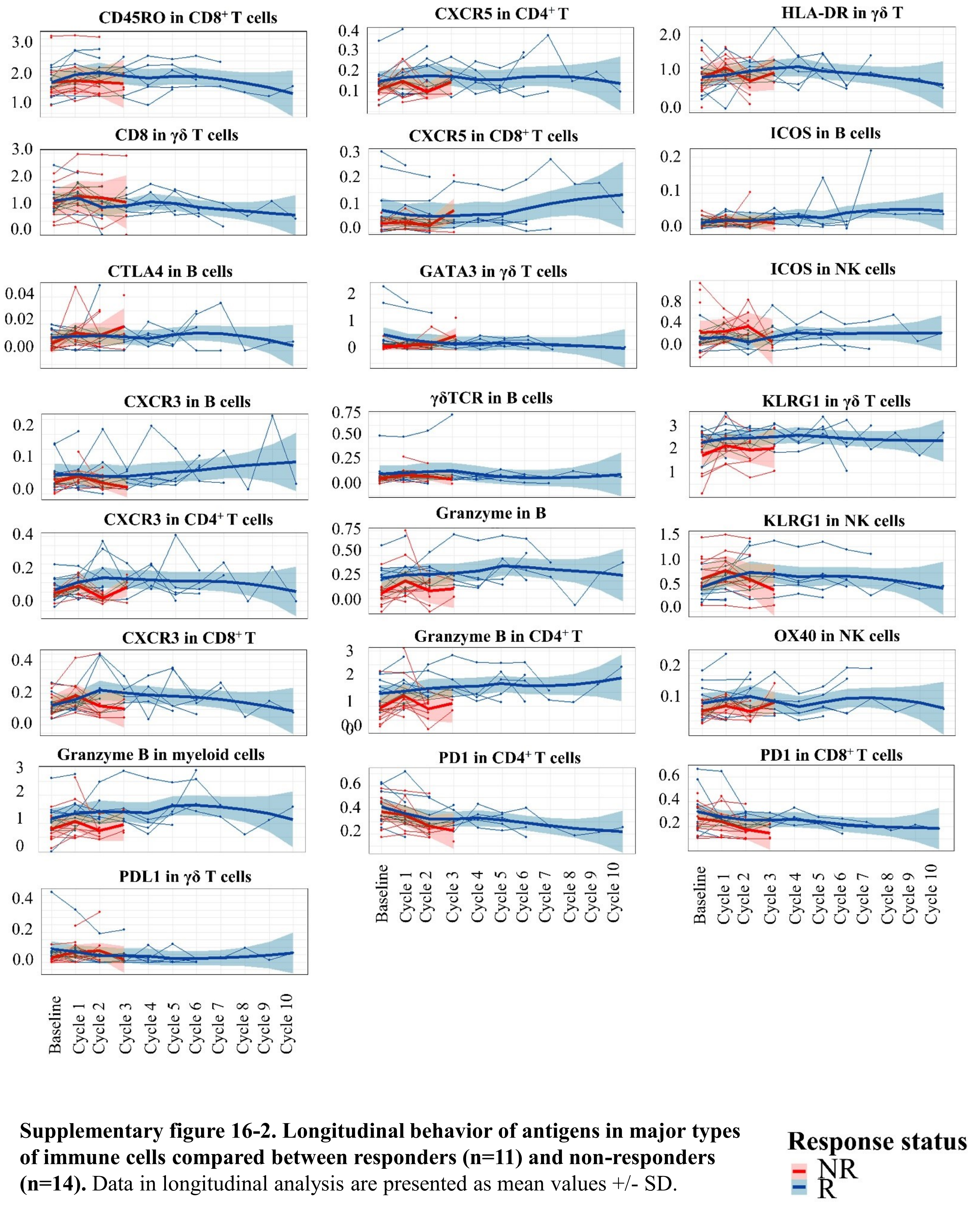

Supplementary figure 16-2. Longitudinal behavior of antigens in major types of immune cells compared between responders (n=11) and non-responders (n=14). Data in longitudinal analysis are presented as mean values +/- SD.

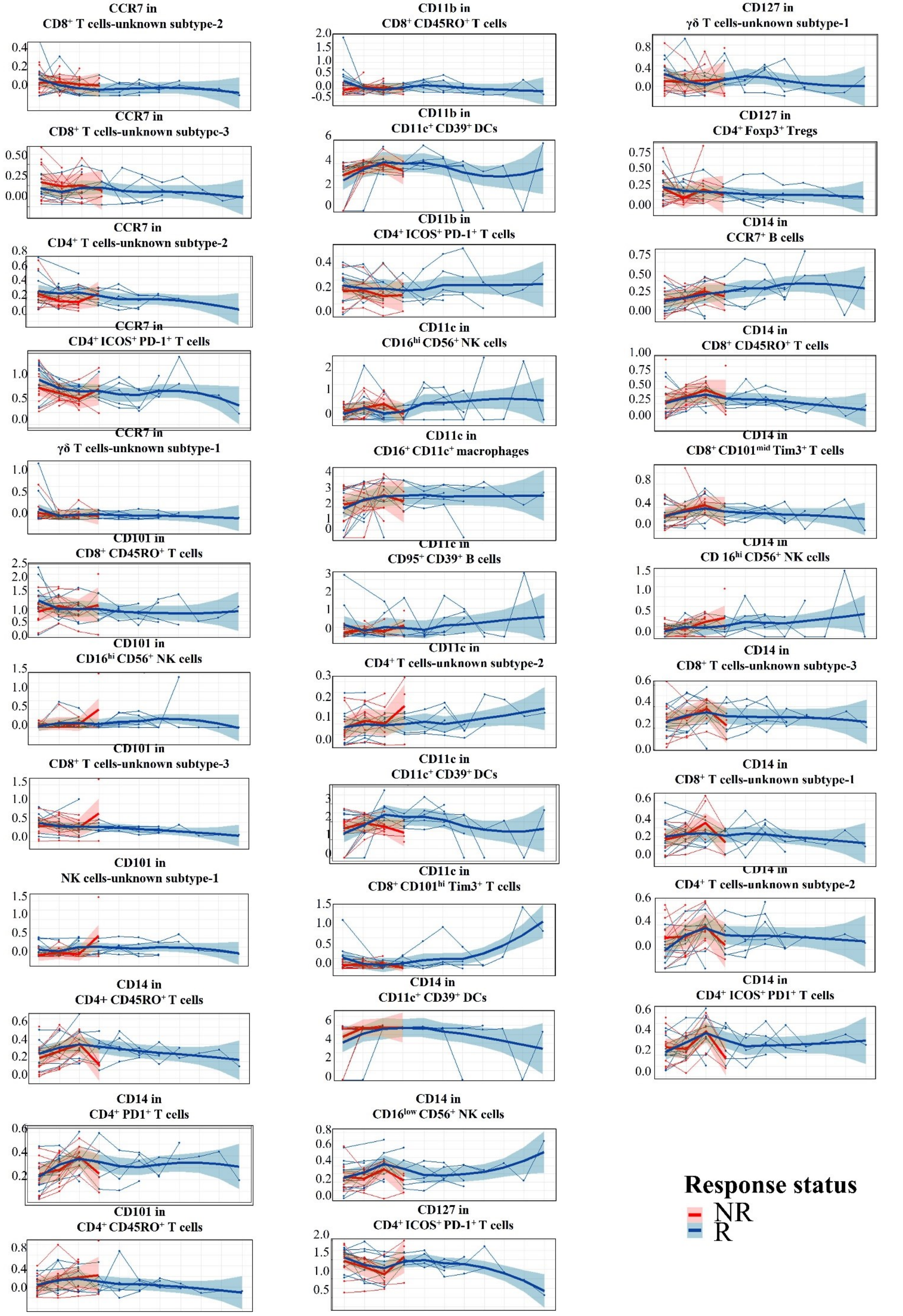

Supplementary figure 17-1. Longitudinal behavior of antigens in different subsets of immune cells compared between responders (n=11) and non-responders (n=14). Data in longitudinal analysis are presented as mean values +/- SD.

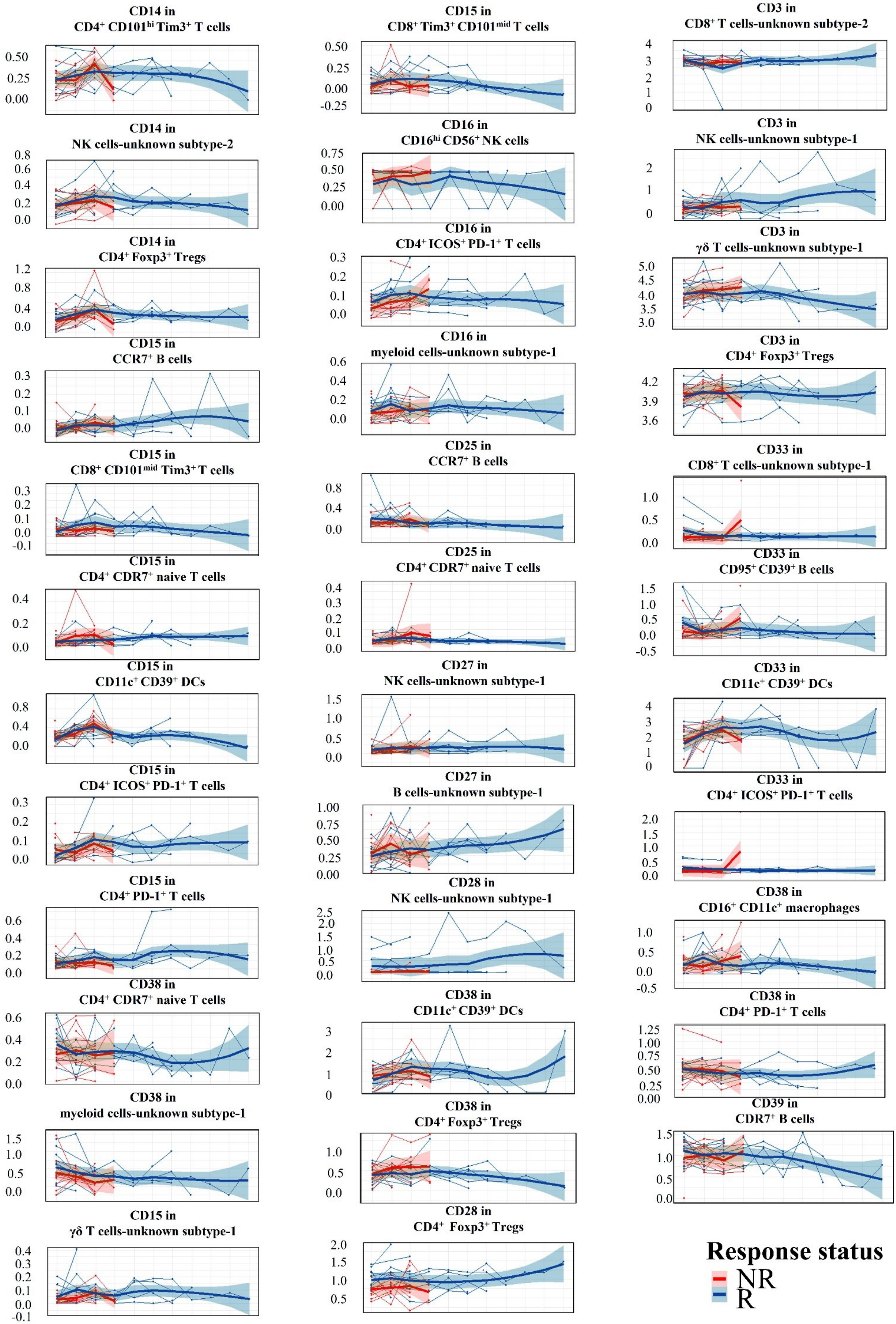

Supplementary figure 17-2. Longitudinal behavior of antigens in different subsets of immune cells compared between responders (n=11) and non-responders (n=14). Data in longitudinal analysis are presented as mean values +/- SD.

Supplementary data

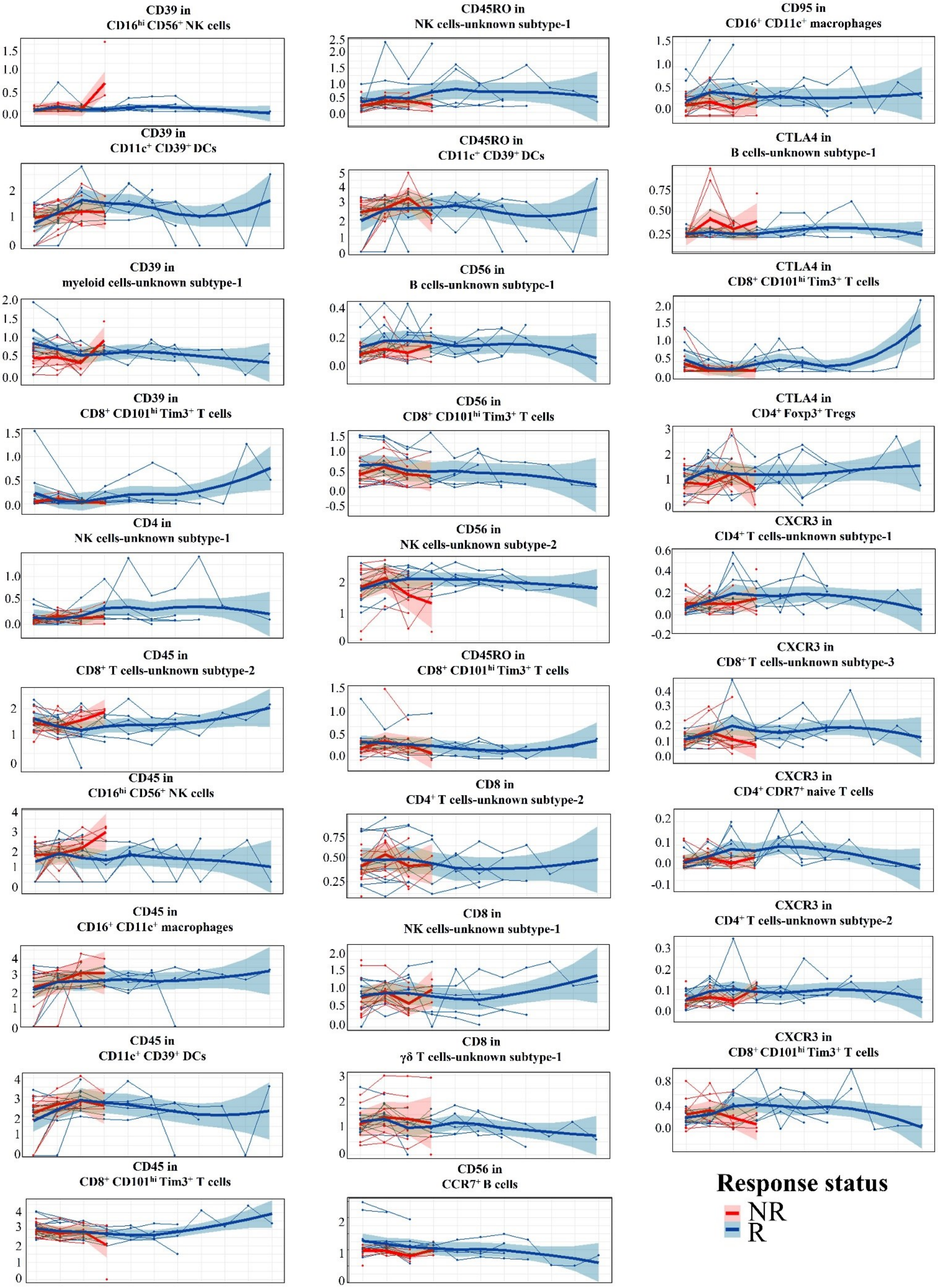

Supplementary figure 17-3. Longitudinal behavior of antigens in different subsets of immune cells compared between responders (n=11) and non-responders (n=14). Data in longitudinal analysis are presented as mean values +/- SD.

Supplementary data

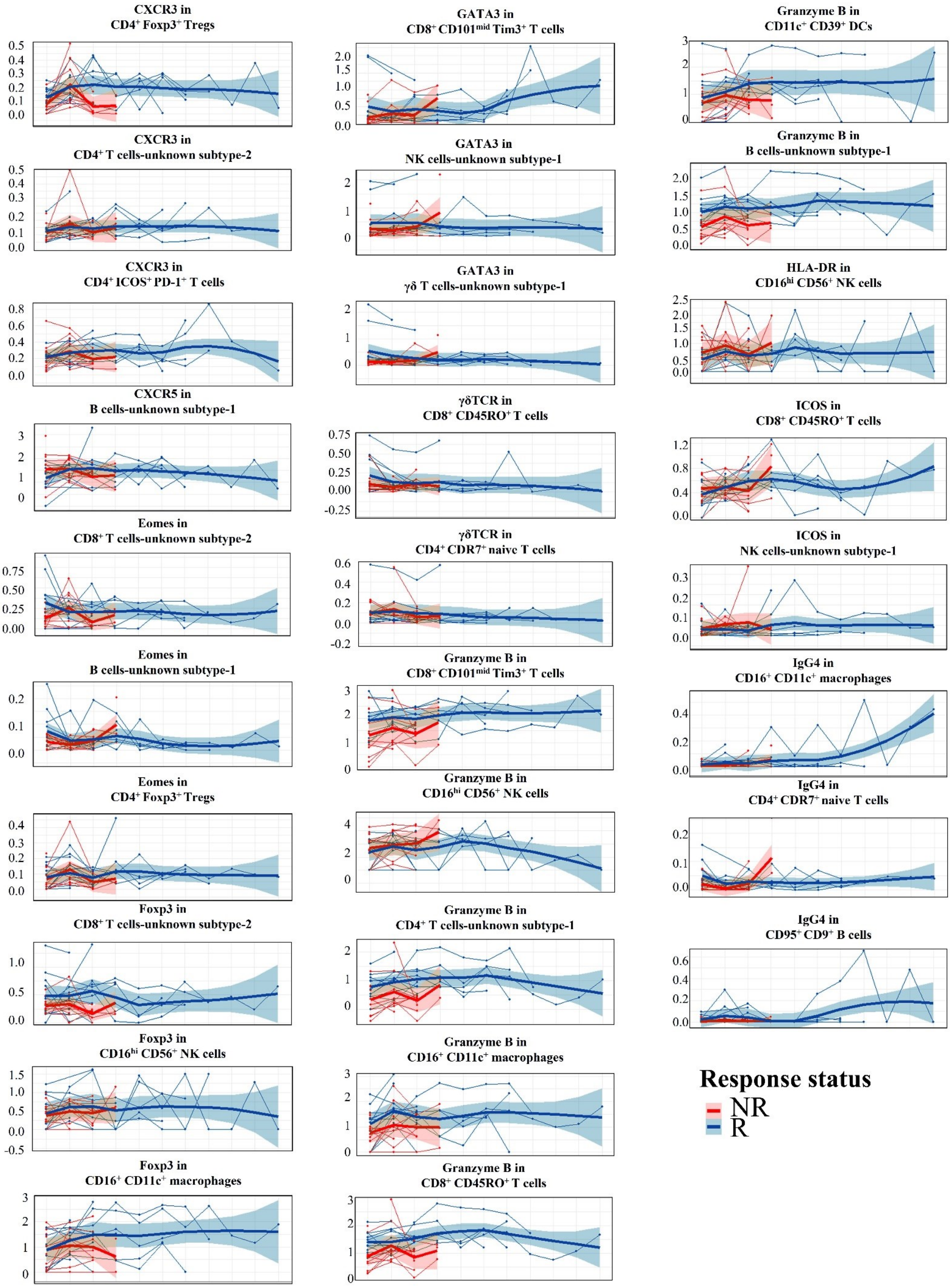

Supplementary figure 17-4. Longitudinal behavior of antigens in different subsets of immune cells compared between responders (n=11) and non-responders (n=14). Data in longitudinal analysis are presented as mean values +/- SD.

Supplementary data

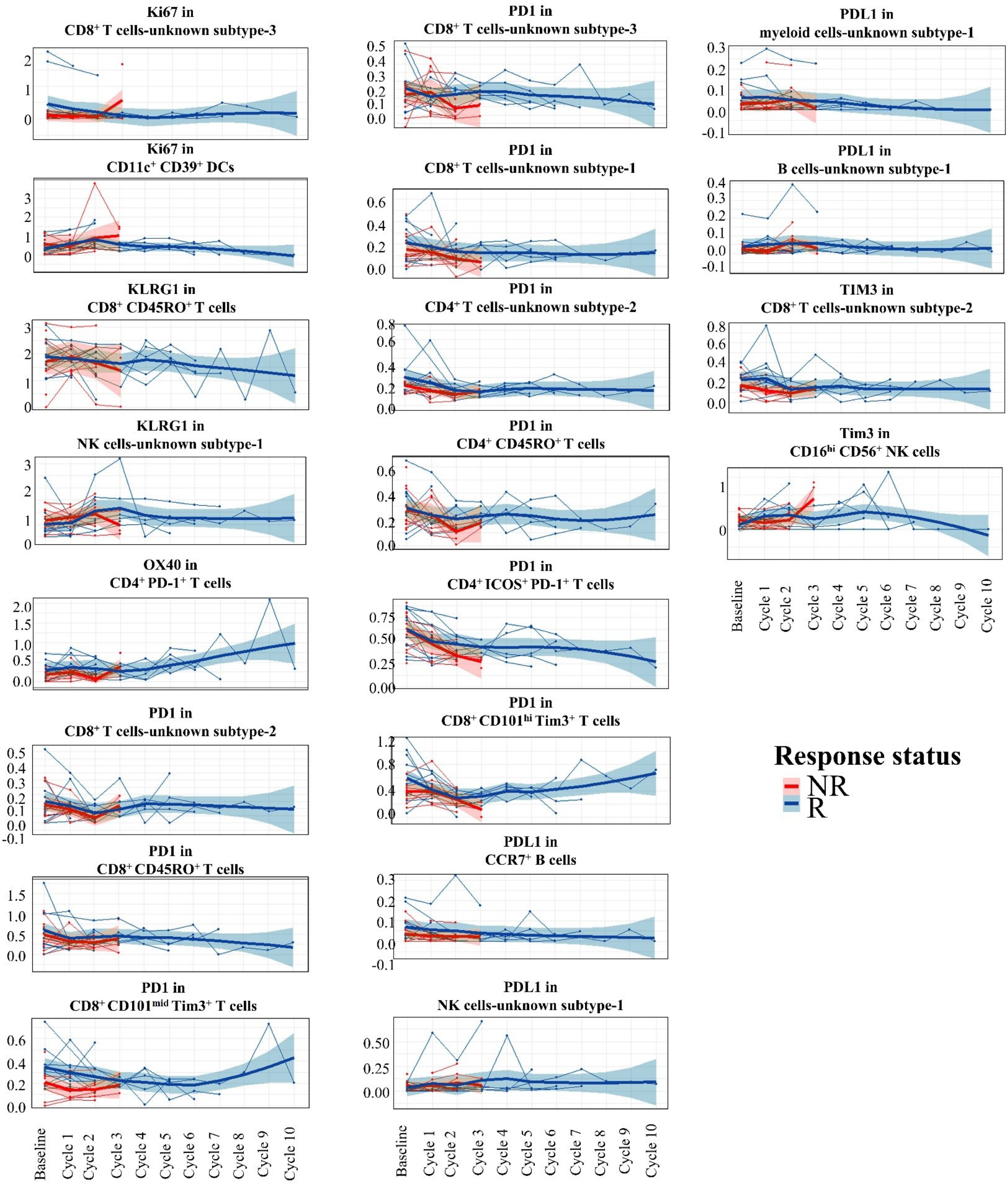

Supplementary figure 17-5. Longitudinal behavior of antigens in different subsets of immune cells compared between responders (n=11) and non-responders (n=14). Data in longitudinal analysis are presented as mean values +/- SD.

Supplementary data

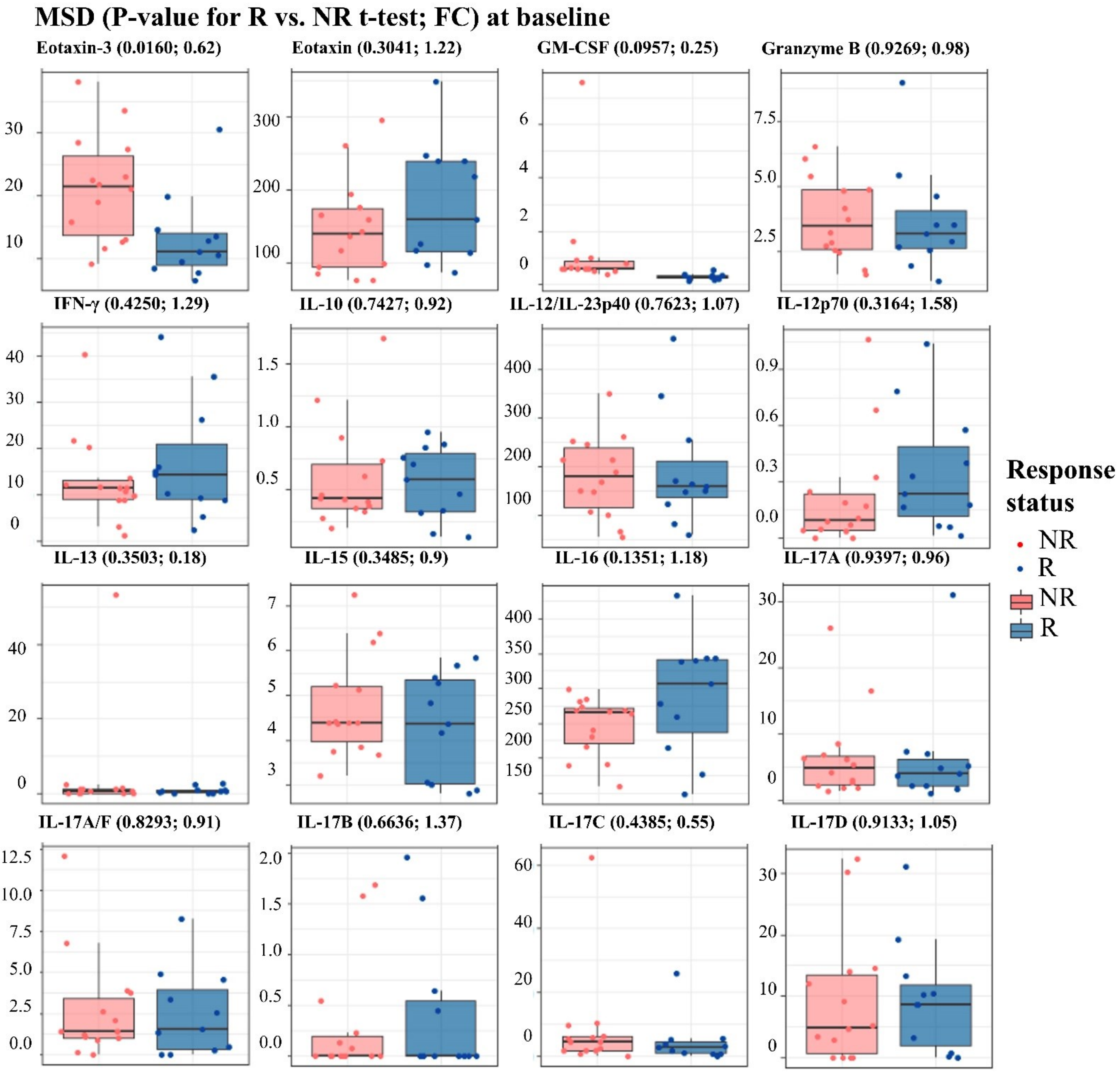

Supplementary figure 18-1. Cytokine levels tested by MSD at baseline and compared between responders (n=11) and non-responders (n=14) in a cohort of NSCLC patients receiving anti-PD-1 treatment. The box plots represent the interquartile range (IQR), with the horizontal line indicating the median. The whiskers extend to the farthest data point within a maximum of 1.5 × IQR. All *p-values* were calculated using two-sided t-tests comparing the R to the NR and were corrected for multiple comparisons using the Benjamini-Hochberg adjustment.

Supplementary data

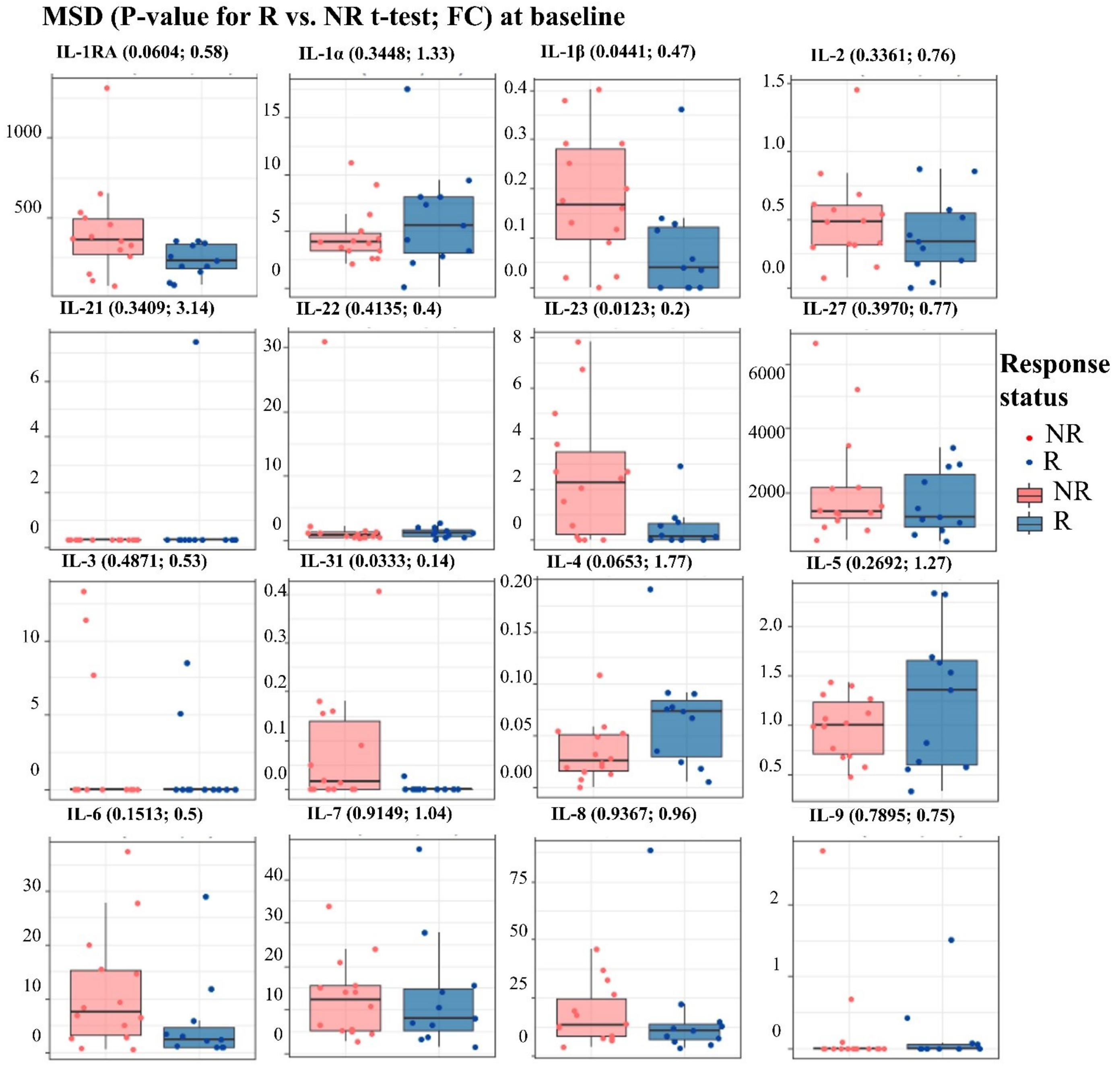

Supplementary figure 18-2. Cytokine levels tested by MSD at baseline and compared between responders (n=11) and non-responders (n=14) in a cohort of NSCLC patients receiving anti-PD-1 treatment. The box plots represent the interquartile range (IQR), with the horizontal line indicating the median. The whiskers extend to the farthest data point within a maximum of 1.5 × IQR. All *p-values* were calculated using two-sided t-tests comparing the R to the NR and were corrected for multiple comparisons using the Benjamini-Hochberg adjustment.

Supplementary data

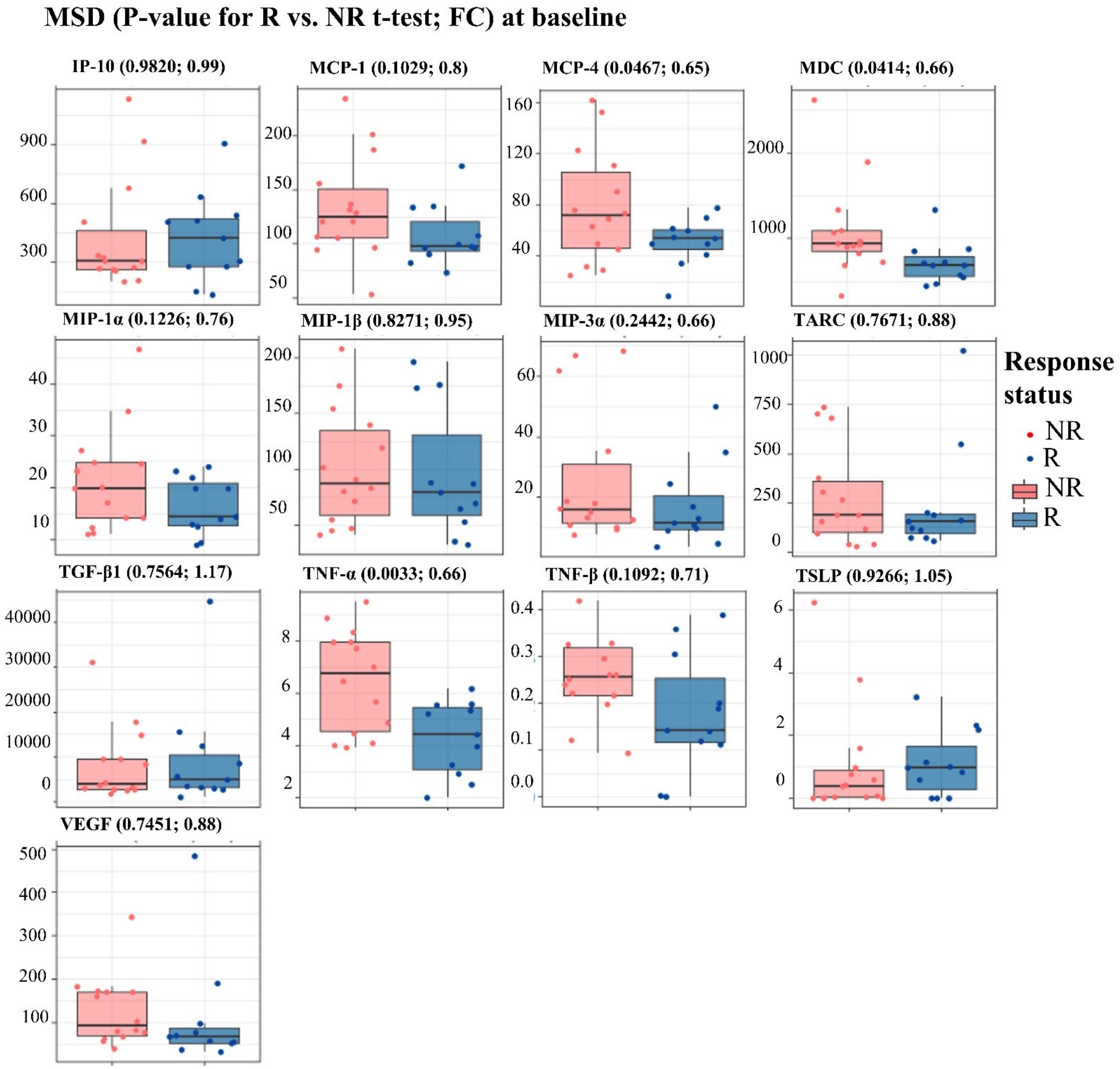

Supplementary figure 18-3. Cytokine levels tested by MSD at baseline and compared between responders (n=11) and non-responders (n=14) in a cohort of NSCLC patients receiving anti-PD-1 treatment. The box plots represent the interquartile range (IQR), with the horizontal line indicating the median. The whiskers extend to the farthest data point within a maximum of 1.5 × IQR. All *p-values* were calculated using two-sided t-tests comparing the R to the NR and were corrected for multiple comparisons using the Benjamini-Hochberg adjustment.

Supplementary data

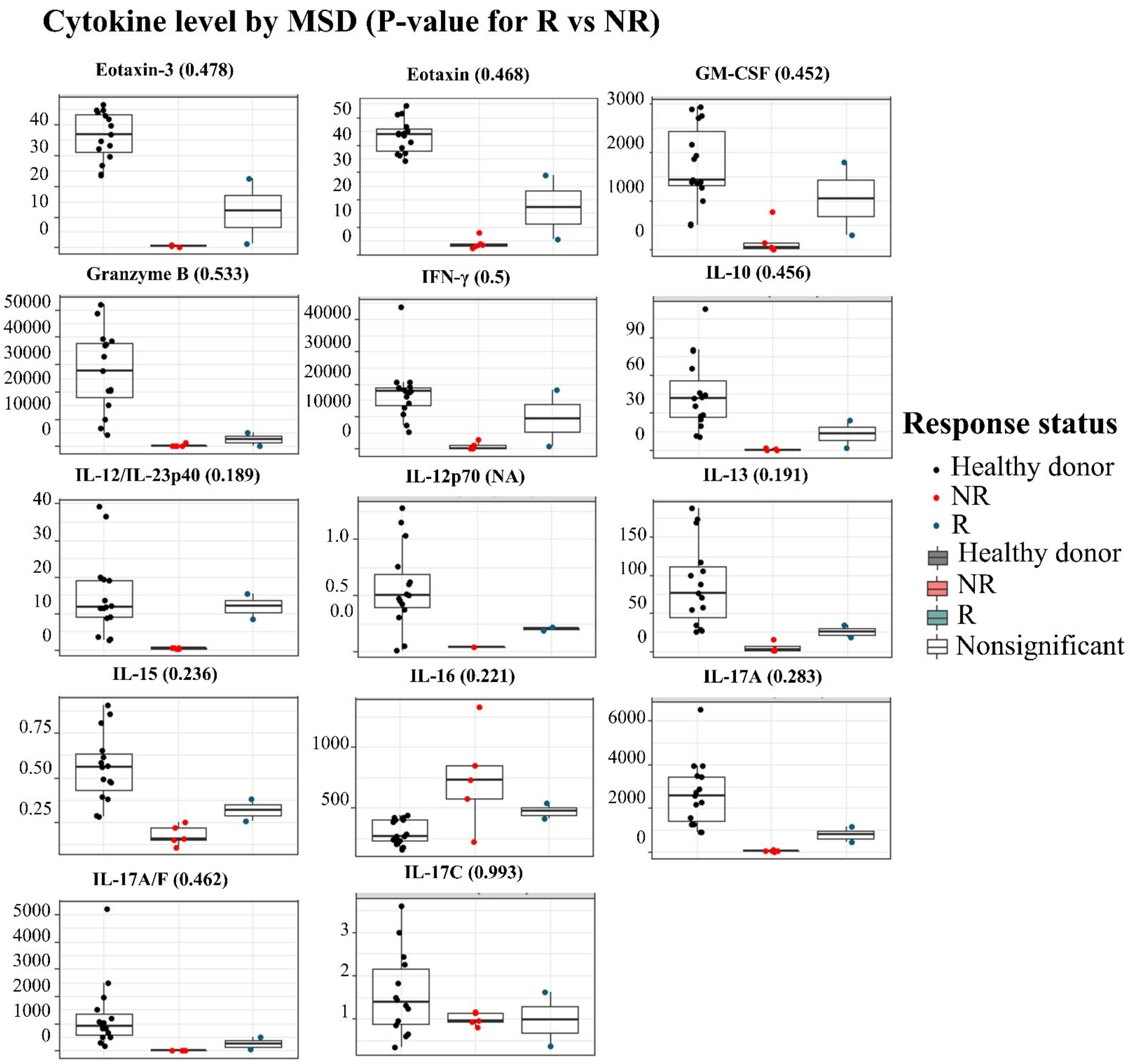

Supplementary figure 19-1. Cytokine levels tested by MSD after T cell stimulation and compared among healthy donors (n=16) and responders (n=11) and non-responders (n=14) in a cohort of NSCLC patients receiving anti-PD-1 treatment. The box plots represent the interquartile range (IQR), with the horizontal line indicating the median. The whiskers extend to the farthest data point within a maximum of 1.5 × IQR. All *p-values* were calculated using two-sided t-tests comparing patients to the HDs and were corrected for multiple comparisons using the Benjamini-Hochberg adjustment.

Supplementary data

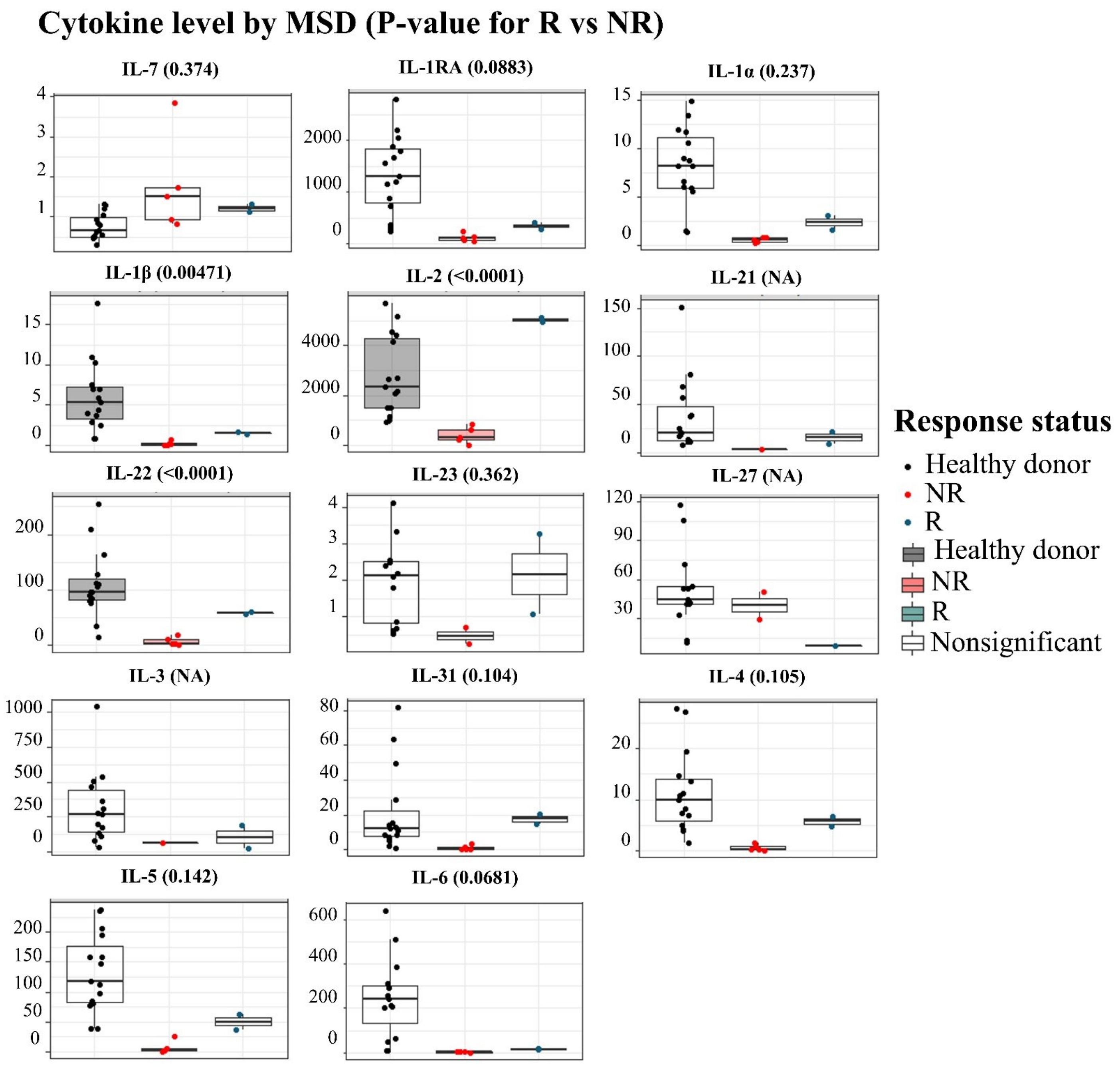

Supplementary figure 19-2. Cytokine levels tested by MSD after T cell stimulation and compared among healthy donors (n=16) and responders (n=11) and non-responders (n=14) in a cohort of NSCLC patients receiving anti-PD-1 treatment. The box plots represent the interquartile range (IQR), with the horizontal line indicating the median. The whiskers extend to the farthest data point within a maximum of 1.5 × IQR. All *p-values* were calculated using two-sided t-tests comparing patients to the HDs and were corrected for multiple comparisons using the Benjamini-Hochberg adjustment.

Supplementary data

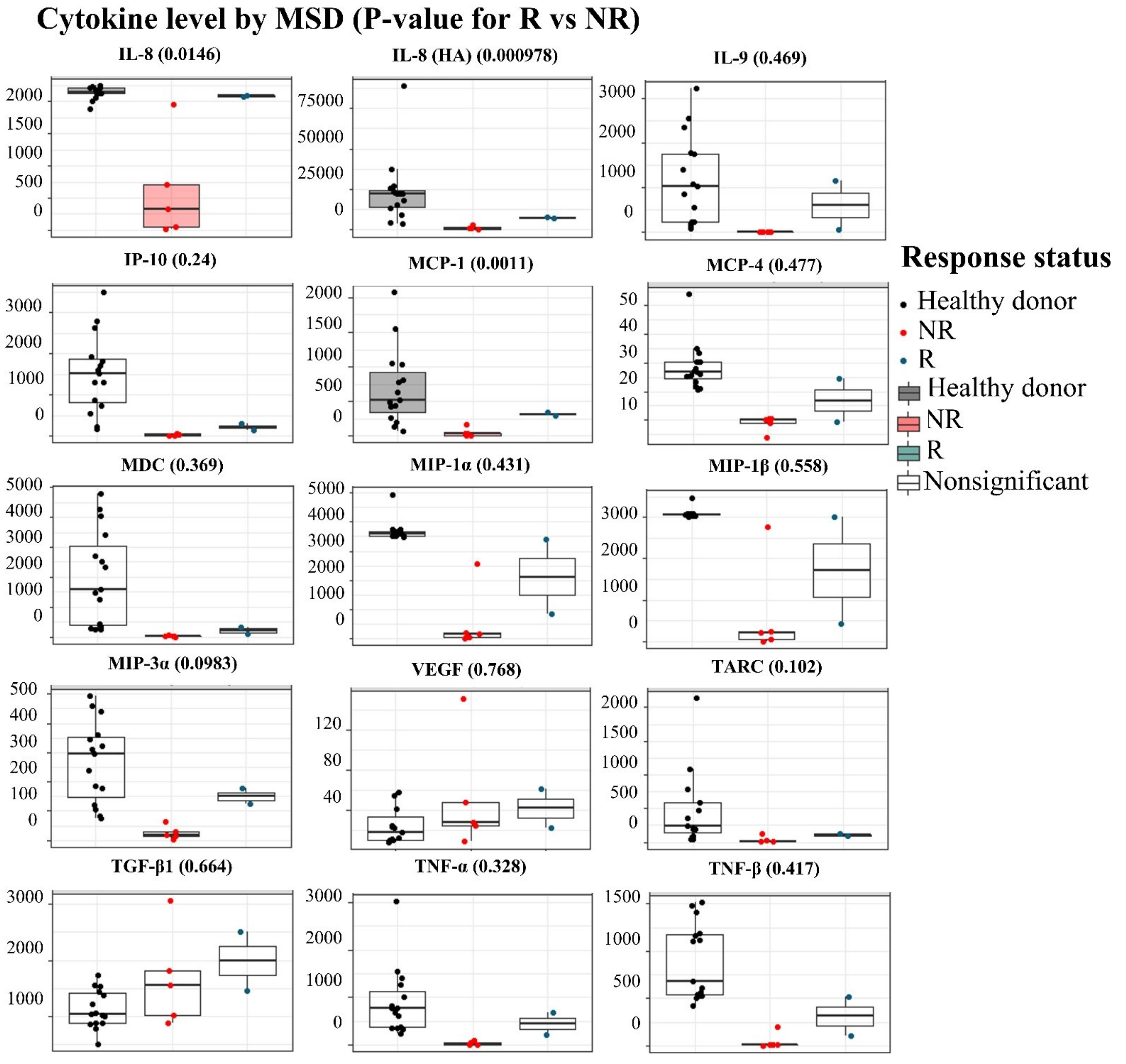

Supplementary figure 19-3. Cytokine levels tested by MSD after T cell stimulation and compared among healthy donors (n=16) and responders (n=11) and non-responders (n=14) in a cohort of NSCLC patients receiving anti-PD-1 treatment. The box plots represent the interquartile range (IQR), with the horizontal line indicating the median. The whiskers extend to the farthest data point within a maximum of 1.5 × IQR. All *p-values* were calculated using two-sided t-tests comparing patients to the HDs and were corrected for multiple comparisons using the Benjamini-Hochberg adjustment.

Supplementary data-Correlations between cytokines and immune cell types

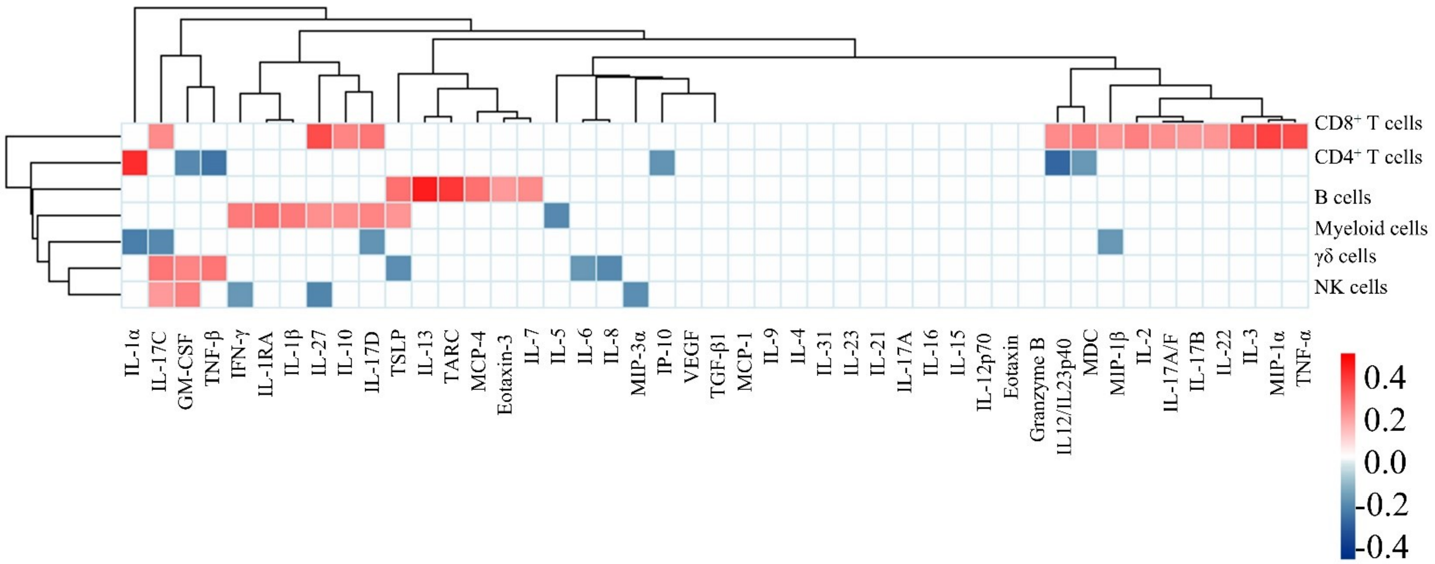

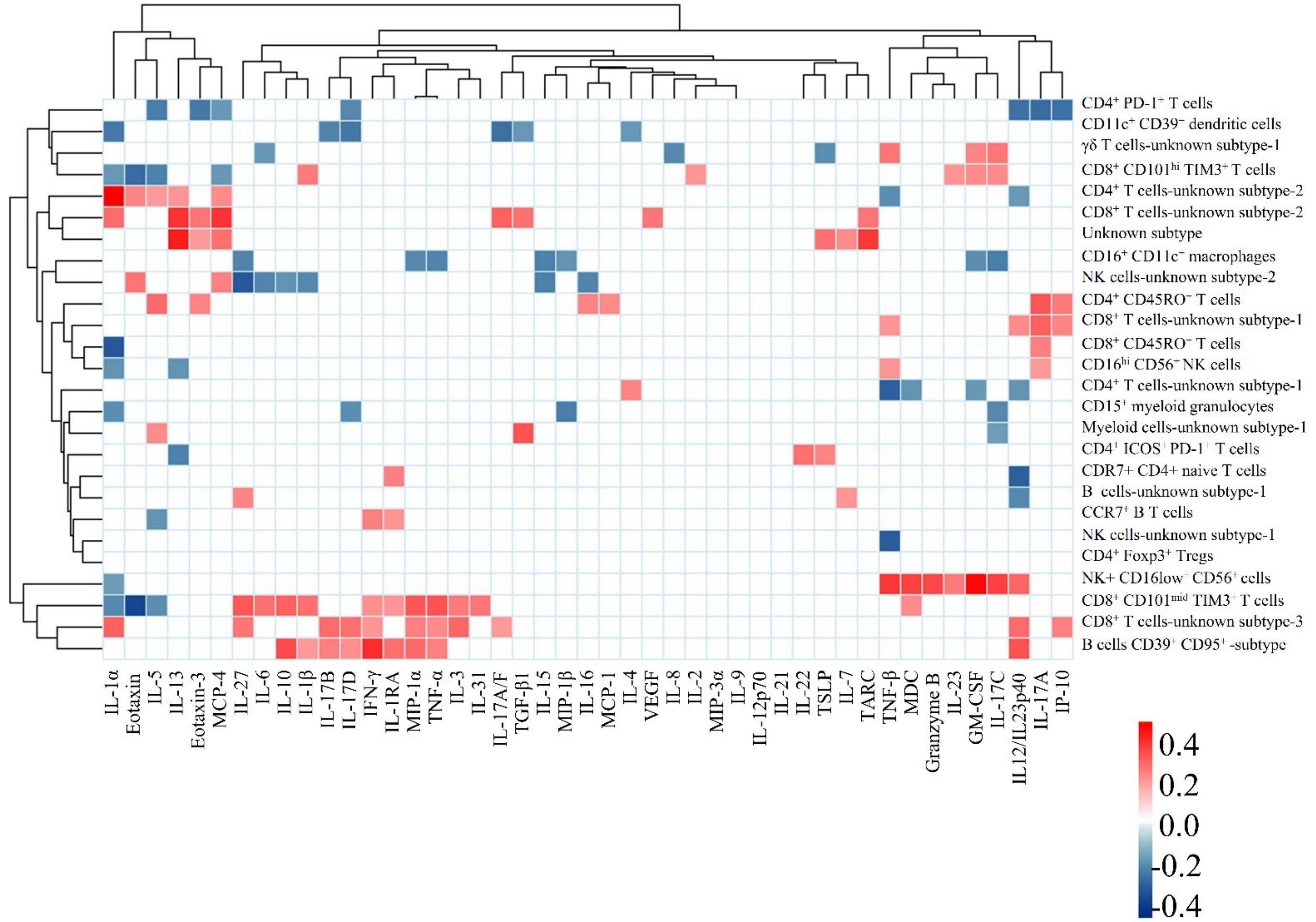

Supplementary figure 20. Heatmap of Pearson correlation coefficient (PCC) values, indicating the correlations between different immune cell types and levels of cytokines in patients. A PCC value larger than 0 indicates a positive relationship. A value less than 0 indicates a negative relationship. If the value equals 0, the correlation between the two objects was not statistically significant.

Supplementary data

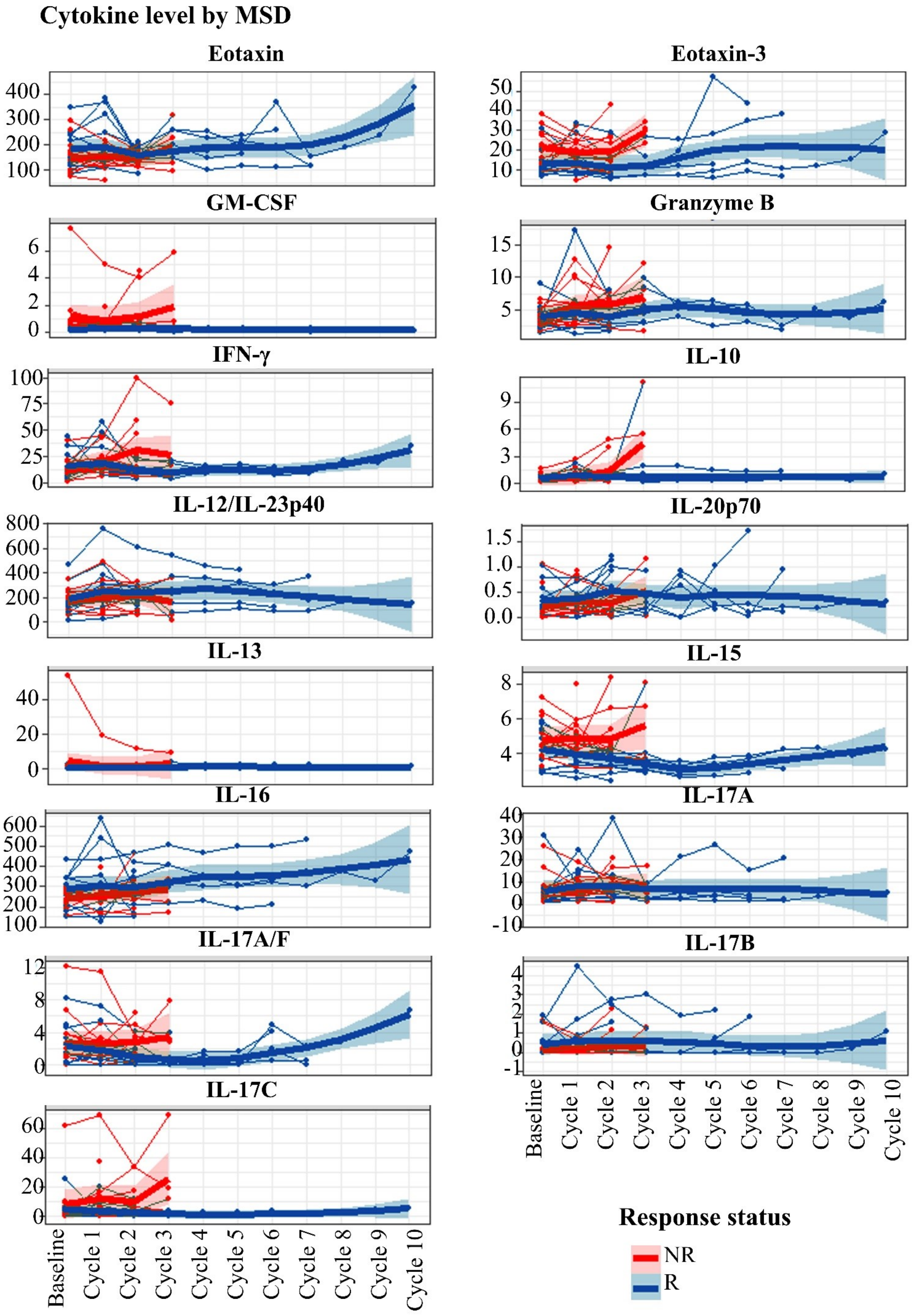

Supplementary figure 21-1. Examples of the longitudinal behavior of cytokines under unstimulated conditions compared between responders and non-responders. Data in longitudinal analysis are presented as mean values +/- SD.

Supplementary data

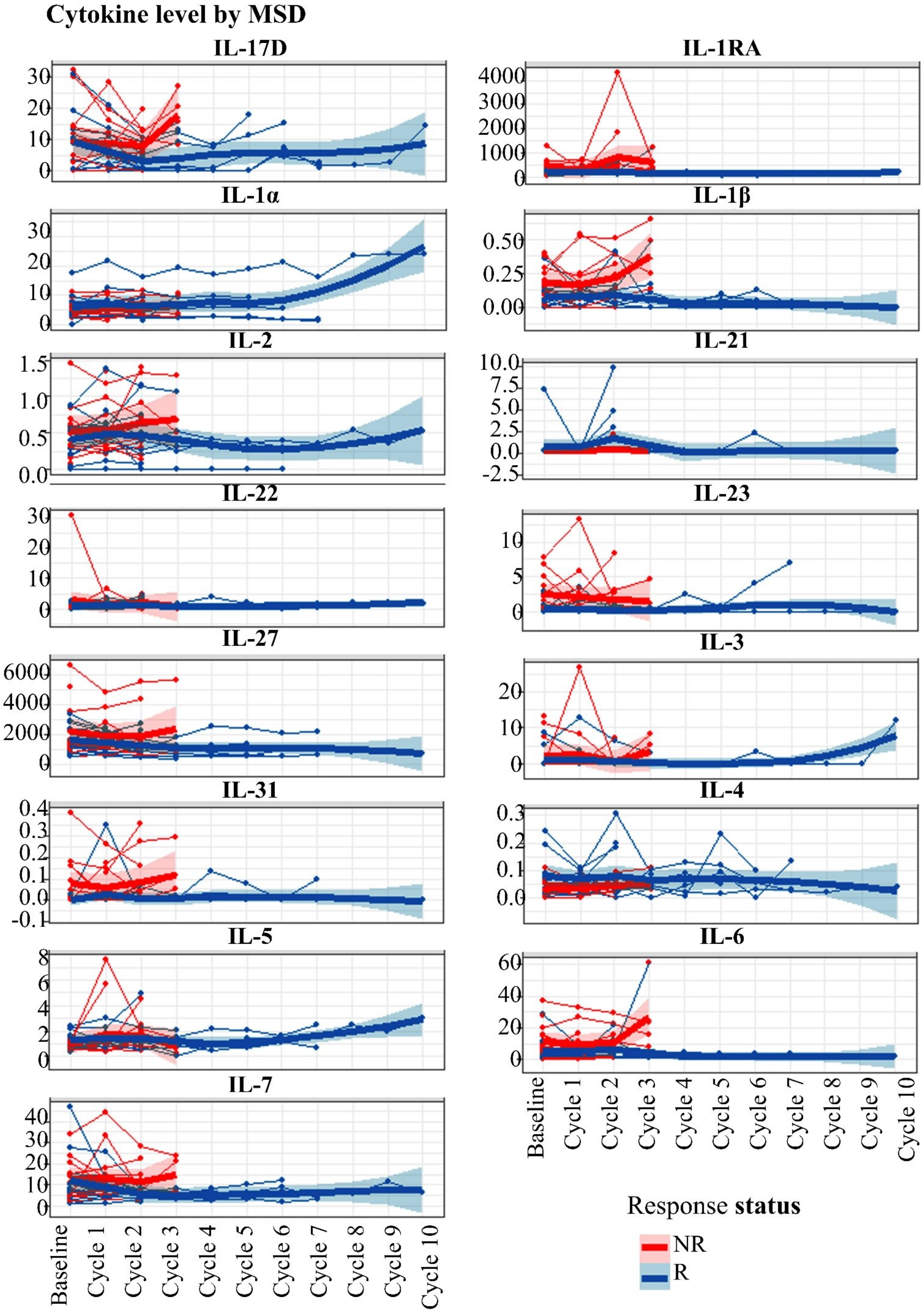

Supplementary figure 21-2. Examples of the longitudinal behavior of cytokines under unstimulated conditions compared between responders and non-responders. Data in longitudinal analysis are presented as mean values +/- SD.

Supplementary data

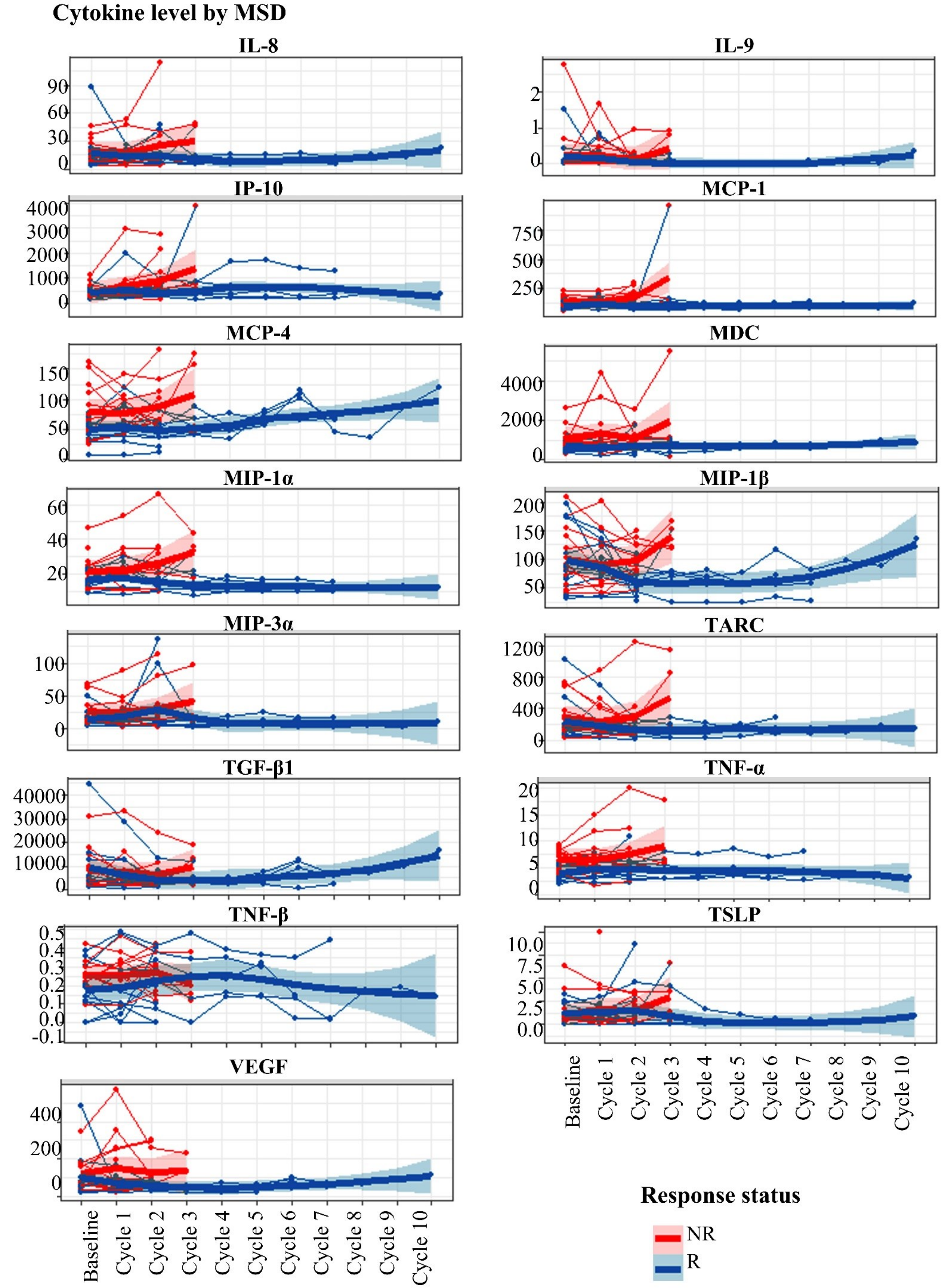

Figure S21-3. Examples of the longitudinal behavior of cytokines under unstimulated conditions compared between responders and non=responders. Data in longitudinal analysis are presented as mean values +/- SD.

Supplementary data

Cytokine level by MSD

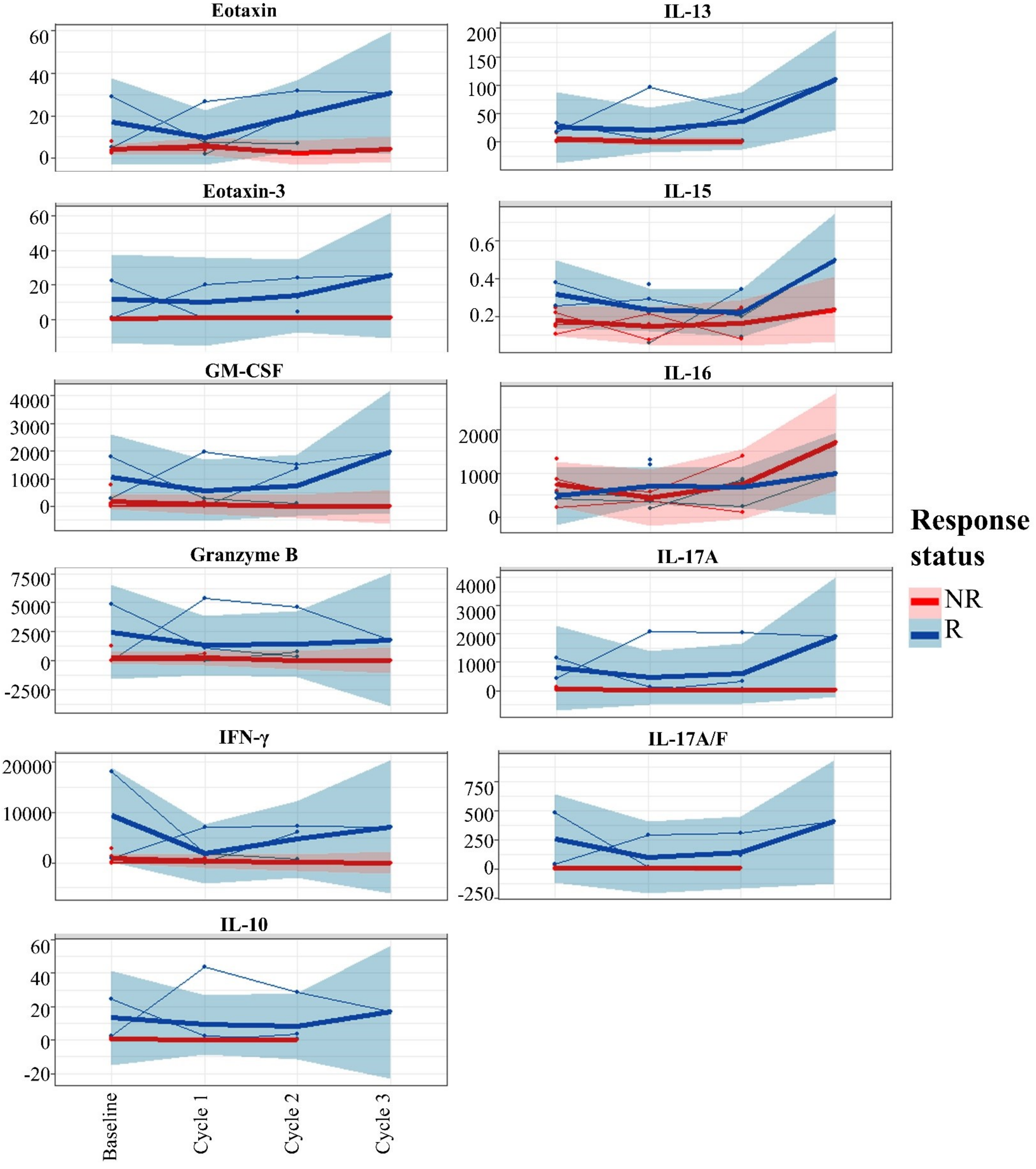

Supplementary figure 22-1. Comparison of the longitudinal behavior of cytokines after stimulation of T cells within the PBMC population of responders or non-responders. Data in longitudinal analysis are presented as mean values +/- SD.

Supplementary data

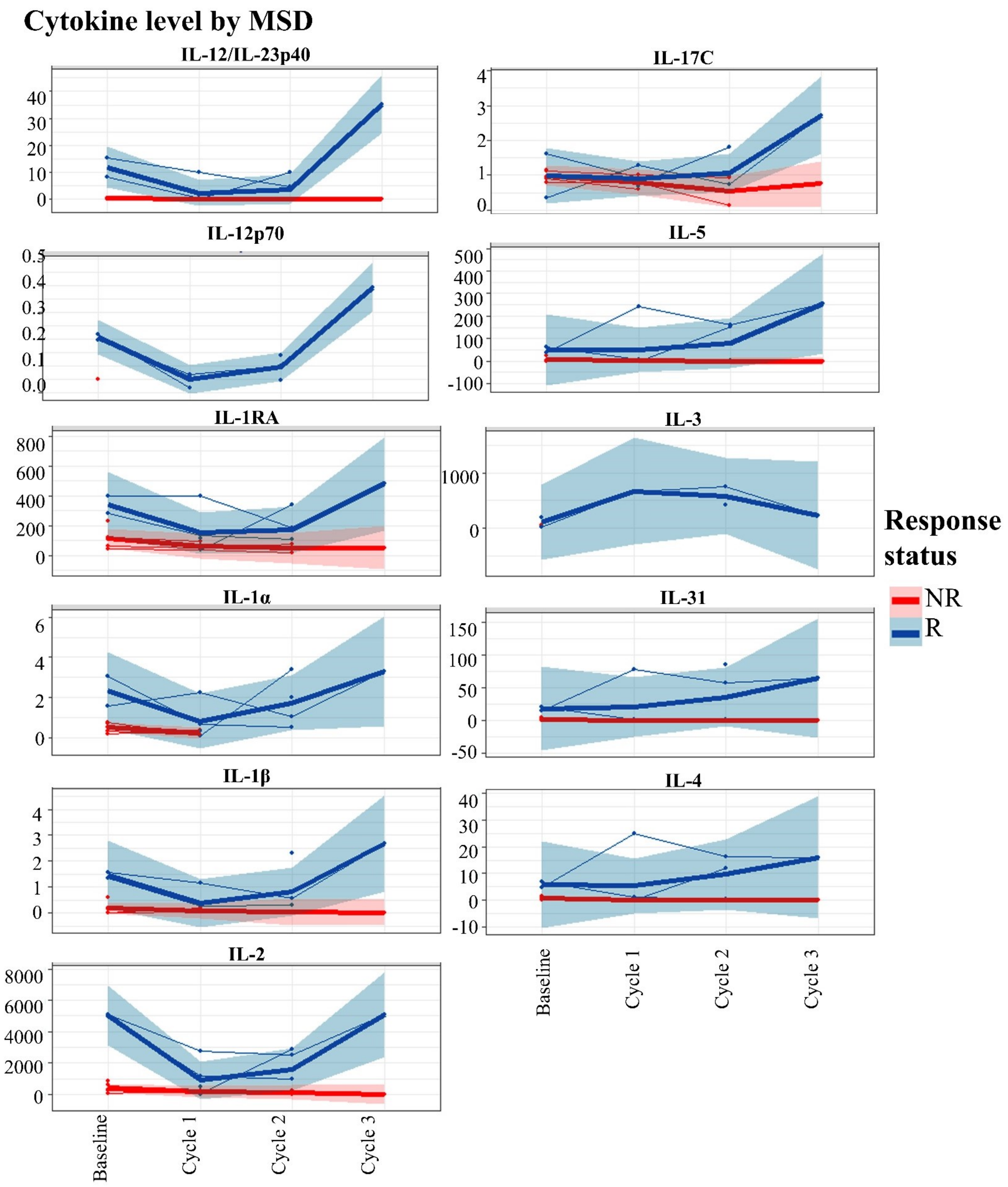

Supplementary figure 22-2. Comparison of the longitudinal behavior of cytokines after stimulation of T cells within the PBMC population of responders or non-responders. Data in longitudinal analysis are presented as mean values +/- SD.

Supplementary data

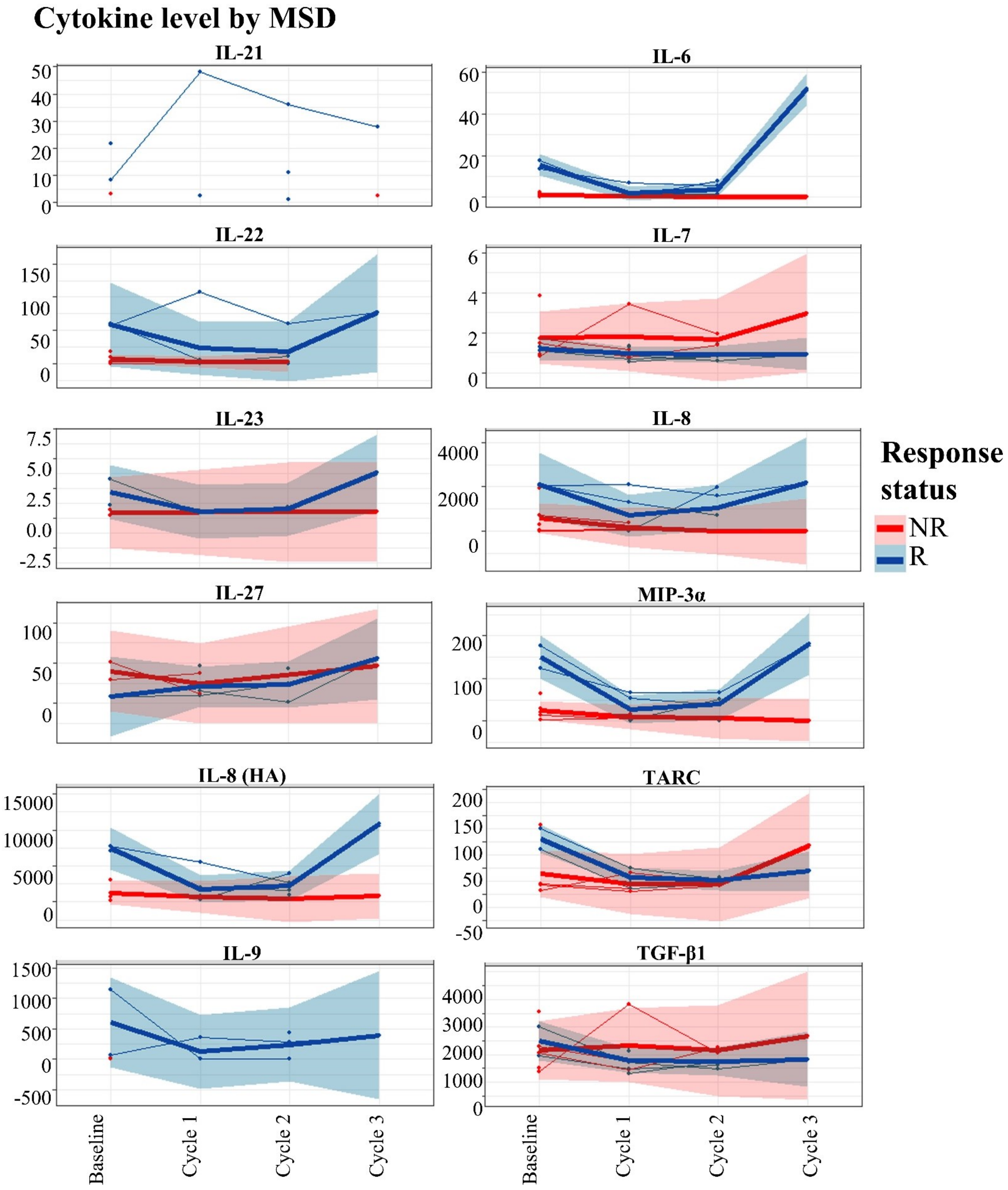

Supplementary figure 22-3. Comparison of the longitudinal behavior of cytokines after stimulation of T cells within the PBMC population of responders or non-responders. Data in longitudinal analysis are presented as mean values +/- SD.

Supplementary data

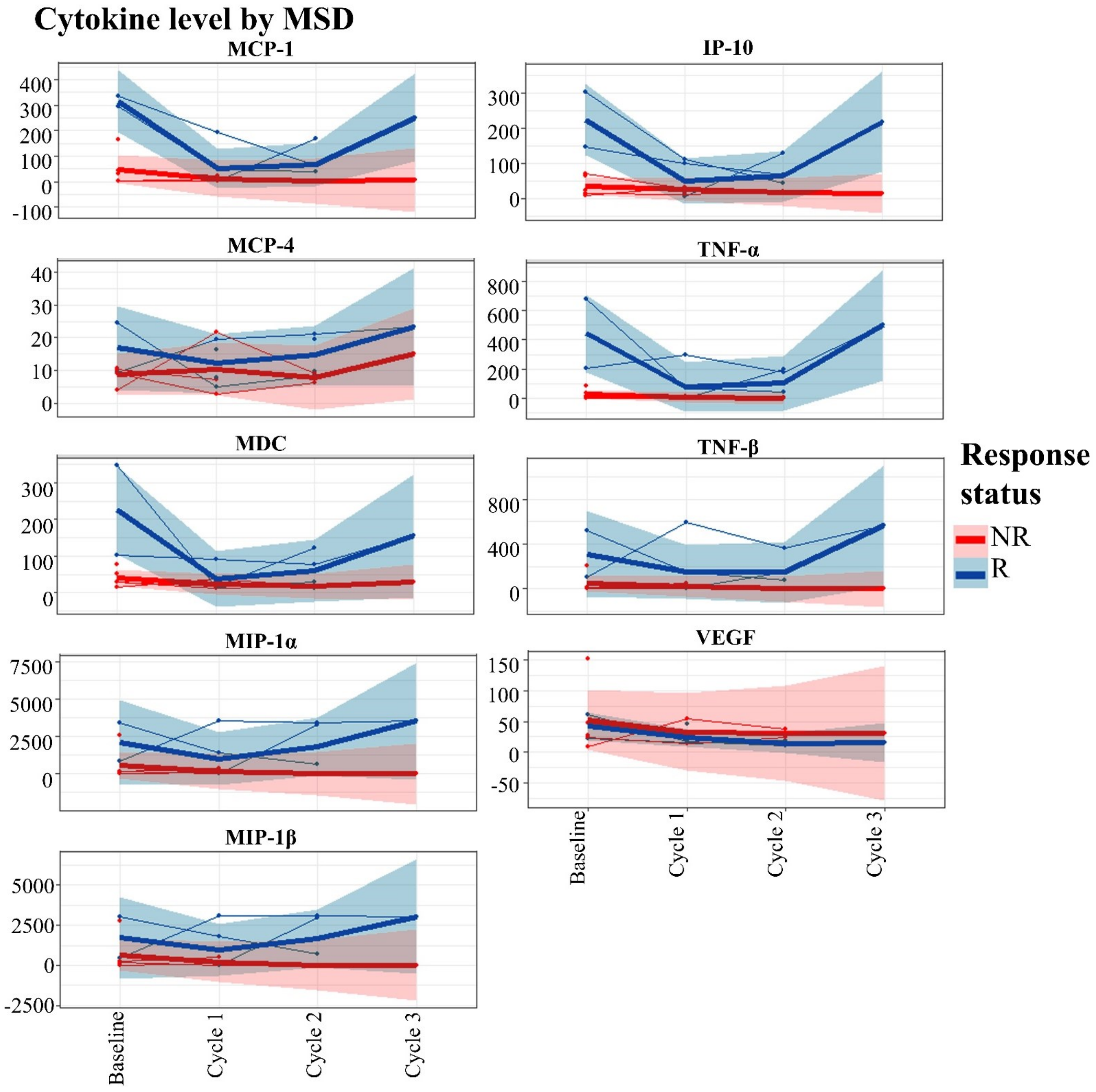

Supplementary figure 22-4. Comparison of the longitudinal behavior of cytokines after stimulation of T cells within the PBMC population of responders or non-responders. Data in longitudinal analysis are presented as mean values +/- SD.

Supplementary data

Characteristic	Patient (N=25)	Responder (N=11)	Non-responder (N=14)
**Sex - no. (%)**			
Male	19 (76)	10 (91)	9 (64)
Female	6 (24)	1 (9)	5 (36)
**Age - mean±sd (min, median, max)**	63.5±12.2 (38, 63, 89)	68.4±11.4 (54, 65, 89)	59.6±11.8 (38, 62, 76)
**Histology - no. (%)**			
Adenocarcinoma	21 (84)	8 (73)	13 (93)
Squamous carcinoma	4 (16)	3 (27)	1 (7)
**Tumor Stage – no. (%)**			
IIIB	1 (4)	1 (9)	0 (0)
IV	24 (96)	10 (91)	14 (100)
**PDL1 IHC level – no. (%)**			
>49%	13 (52)	6 (55)	7 (50)
1%~49%	3 (12)	2 (18)	1 (7)
<1%	8 (32)	3 (27)	5 (36)
N/A	1 (4)	0 (0)	1 (7)
**Duration of response (in month) - mean±sd (min, median, max)**	6.2±4.5 (0, 5, 15)	9.4±3.5 (4, 10, 15)	3.6±3.6 (0, 3, 15)

Supplementary table 1. Summary of the clinical evaluation results for all the patients (n=25) after receipt of anti-PD1 treatment.

Supplementary data

			PDL1 IHC level			
			>49%	1-49%	<1%	Total
Response state	Responder	Count	6	2	3	11
		Expected Count	6.0	1.4	3.7	11.0
		In response state%	54.5%	18.2%	27.3%	100.0%
	Non-responder	Count	7	1	5	13
		Expected Count	7.0	1.6	4.3	13.0
		In response state%	53.8%	7.7%	38.5%	100.0%
Total		Count	13	3	8	24
		Expected Count	13.0	3.0	8.0	24.0
		In response state%	54.2%	12.5%	33.3%	100.0%

	Value	df	Asymptotic Significance (2-sided)
Pearson Chi-Square	0.749	2	0.688

Supplementary table 2. Analysis of the relevance and dependency between PD-L1 IHC level and the response statue of immunotherapy.

Supplementary data

			TMB		
			High	Low	Total
Response state	Responder	Count	1	7	8
		Expected Count	1.1	6.9	8.0
		In response state%	12.5%	87.5%	100.0%
	Non-responder	Count	1	6	7
		Expected Count	0.9	6.1	7.0
		In response state%	14.3%	85.7%	100.0%
Total		Count	2	13	15
		Expected Count	2.0	13.0	15.0
		In response state%	13.3%	86.7%	100.0%

	Value	df	Asymptotic Significance (2-sided)
Pearson Chi-Square	0.01	1	0.919

Supplementary table 3. Analysis of the relevance and dependency between TMB and the response statue of immunotherapy.

Supplementary data

Metal	Antibody	Clone	Provider	Dilution (x-fold)
Y-89	CD45	HI30	Fluidigm	100
Cd-112/114	CD14	TUK4	Invitrogen	100
ln-115	CD15	HI98	Biolegend	100
Ce-140	Anti-PE	PE001	Biolegend	100
	TCRgt-PE	5A6.E9	Invitrogen	25
Pr-141	CD56	NCAM16.2	BD	100
Nd-142	CD19	HIB-19	Biolegend	100
Nd-143	CD27	LG.7F9	Biolegend	100
Nd-144	CD3	UCHT1	bl300414	100
Nd-145	CD8	SK1	Biolegend	100
Nd-146	HLA-DR	L243	Biolegend	100
Sm-147	CD4	SK3	Biolegend	100
Nd-148	CD45RO	UCHL1	Biolegend	50
Sm-149	ICOS	C398.4A	Biolegend	50
Nd-150	Granzyme B	CLB-GB11	Abcam	800
Eu-151	CD69	FN50	Biolegend	100
Sm-152	CD101	BB27	eBioscience	100
Eu-153	KLRG1	13F2F12	eBioscience	100
Sm-154	CXCR5	RF8B2	BD	100
Gd-155	CD33	WM53	Biolegend	100
Gd-156	Tbet	4B10	Biolegend	100
Gd-157	CXCR3	MAB160	R&D	100
Gd-158	SAV	NA	in-housse	NA
	FOXP3-biot	PCH101	eBioscience	100
Tb-159	PDL1	29E.2A3	Biolegend	25
Gd-160	PD-1	eBioJ105	eBioscience	50
Dy-161	TIM-3	MAB2365	R&D	100
Dy-162	CD95	DX2	Biolegend	100
Dy-163	CD127	AO19D5	Biolegend	100
Dy-164	IgG4	Fc-UNLB	Southern Biotech	100
Ho-165	Ki67	B56	BD	100
Er-166	Ox40	MAB3388	R&D	50
Er-167	GATA3	TWAJ	eBioscience	100
Er-168	CCR7	MAB197	R&D	50
Tm-169	CD25	M-A251	Biolegend	100
Er-170	CTLA4	14D3	eBioscience	25
Yb-171	CD28	CD28.2	Biolegend	50
Yb-172	CD38	HIT2	Biolegend	100
Yb-173	CD39	A1	Biolegend	100
Yb-174	Eomes	WD1928	eBioscience	50
Lu-175	CD11c	B-Ly6	BD	100
Yb-176	CD11b	ICRF44	Biolegend	100
Bi-209	CD16	3g8	Fluidigm	100

Supplementary table 4. Composition of the 40-target panel for CyTOF tests.

Supplementary methods

1. Methodological verification

(1) Validation of the CyTOF results by FACS.

Table. The designed FACS panels

Fluorochrome (MUST Aria III)	Panel 1 (T cell proliferation)	Panel 2 (T cell proliferation, CD38 and PD-1)	Panel 3 (MDSC and CD38)	Panel 4 (Breg and CD38)	Panel 5 (Treg, CD38 and CD101)
	Require intra-cellular staining	Require intra-cellular staining	Only surface staining	Only surface staining	Only surface staining
FITC	Ki-67	Ki-67	CD45	CD45	CD45
PE	CD4	CD38	CD38	CD38	CD38
PerCP	CD3	CD3	CD15	CD19	CD3
PE-Cy5					
PE-CF594	CD8a	CD8a	HLA-DR		CD4
PE-Cy7	CD45	CD45	CD14		CD25
APC	CD45RA	PD-1	CD33	CD24	CD101
APC-Cy7	Fix/Viable NIR2	Fix/Viable NIR2	Fix/Viable NIR2	Fix/Viable NIR2	Fix/Viable NIR2

Three PBMC samples from healthy donors were tested by both FACS and CyTOF. The results showed there was no statistical significance between these two methods suggesting the methodology of CyTOF was validated.

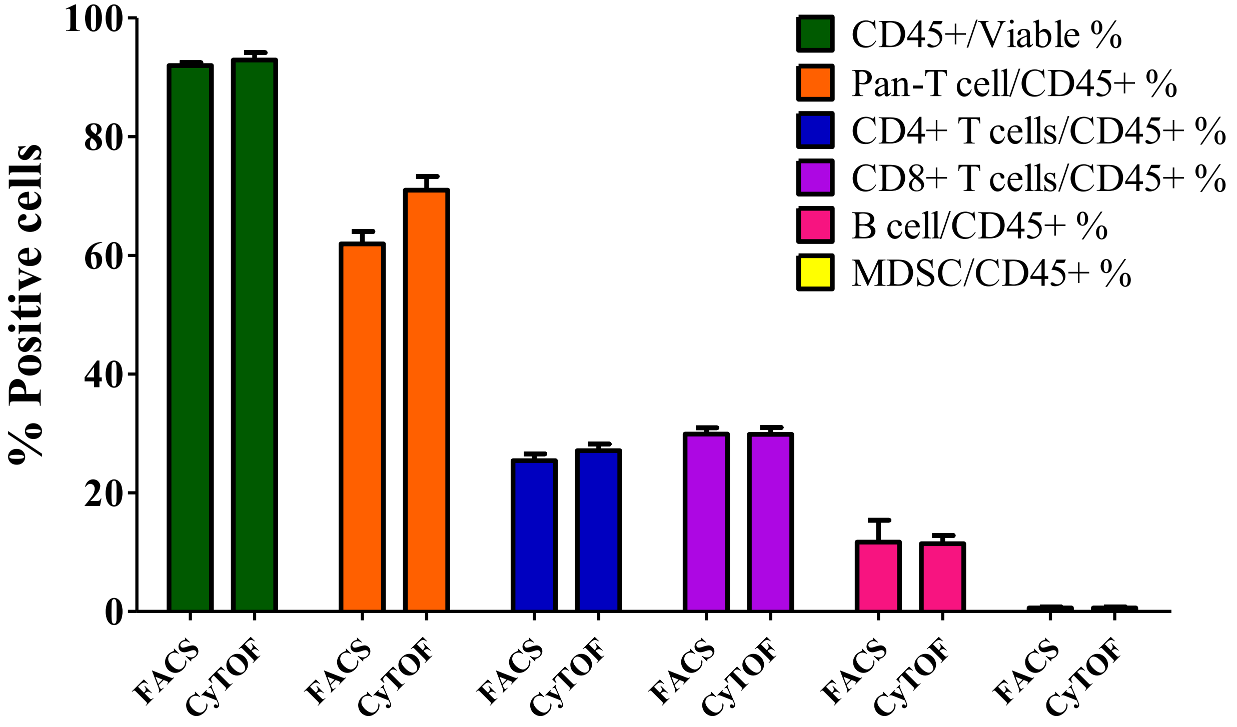

(2) Test of the proportion of viable cells recovered after cryopreservation.

The recovery rates were all over 80% meeting the demanding number of cells required for CyTOF detection

HD	Frozen PBMC	Viable PBMC after thawing	Dead cells	Recovery rate
HD-1	7.50E+06	7.30E+06	<1E+06	97.33%
HD-2	7.50E+06	6.10E+06	<1E+06	81.33%
HD-3	7.50E+06	6.80E+06	<1E+06	90.67%

(3) Test of the influence of time on the results.

A comparative experiment was conducted on the PBMC of healthy people after a long time of storage.

The results showed that there was no significant change in the results after a long time of storage as shown below.

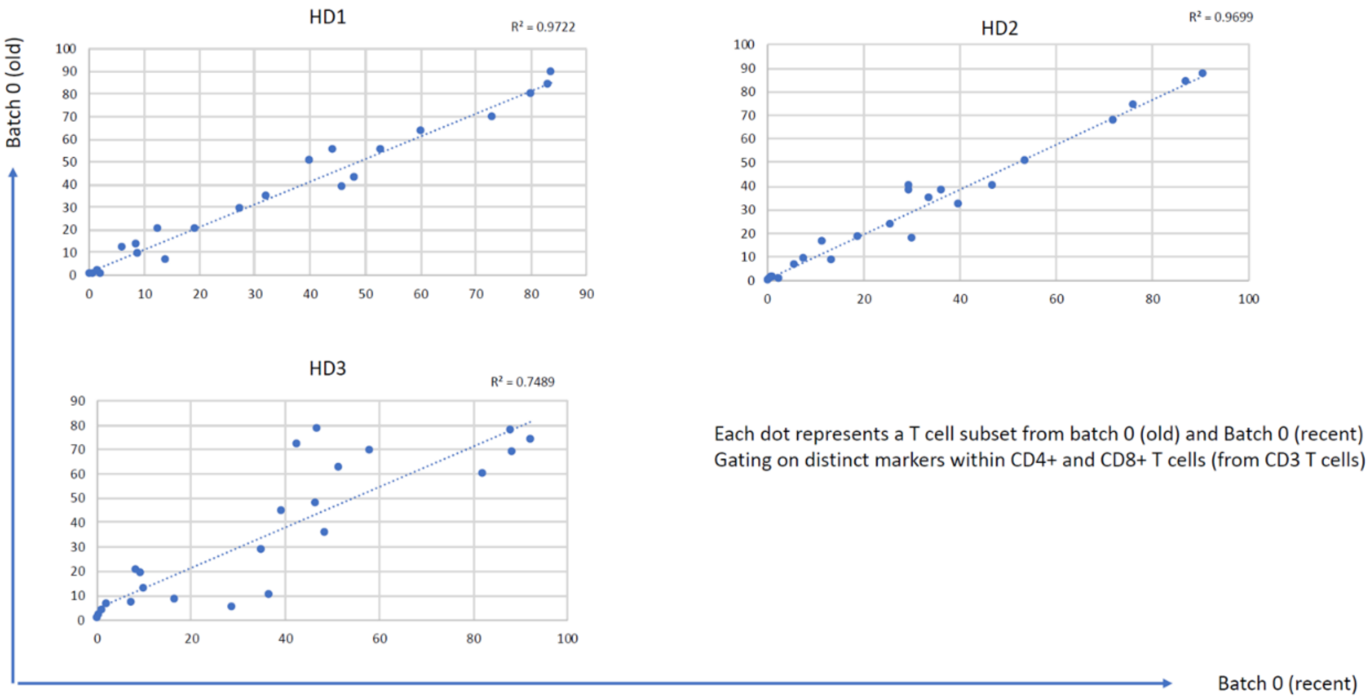

The monitoring test on different batches was also conducted.

The results showed that the frequency of different immune populations from different batches were basically consistent.

2. CyTOF QC test:

If the viable cells count of the samples were lower than 1000 or if the sample is the outlier in principle component analysis (PCA) results (as shown below), it means the samples failed the CyTOF QC test and were excluded from further investigations.

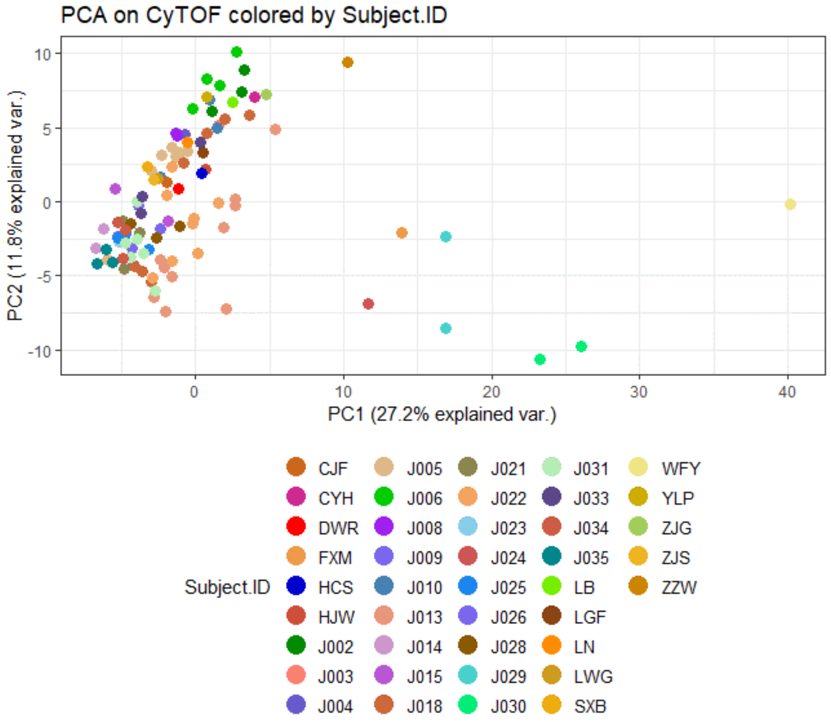

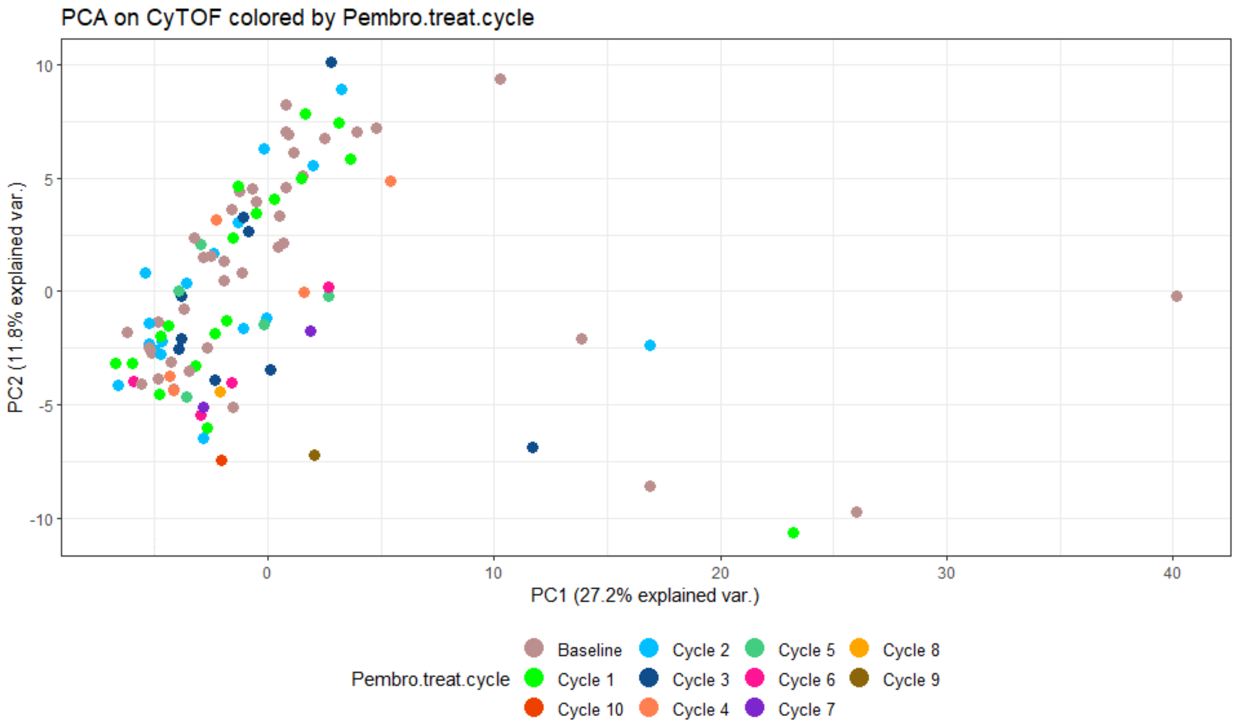

3. Gating strategies

FACS panel (lymphoid)

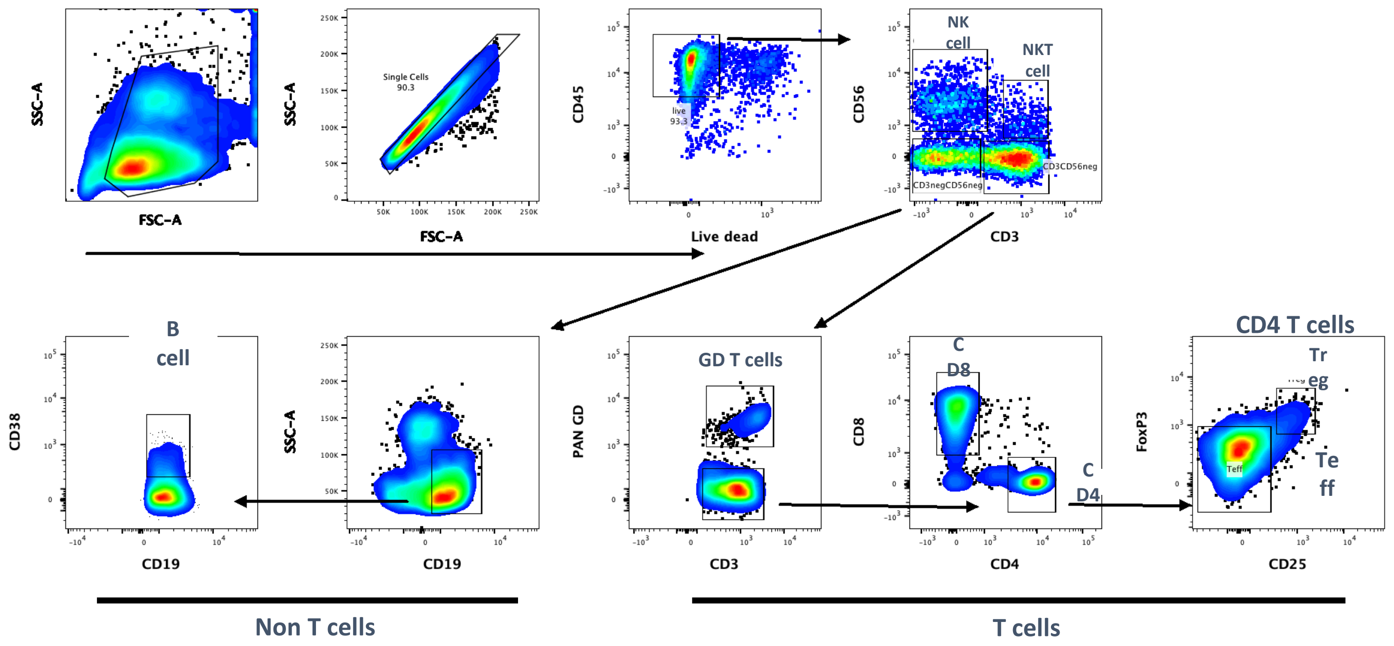

Represented gating values

J008A (NR)

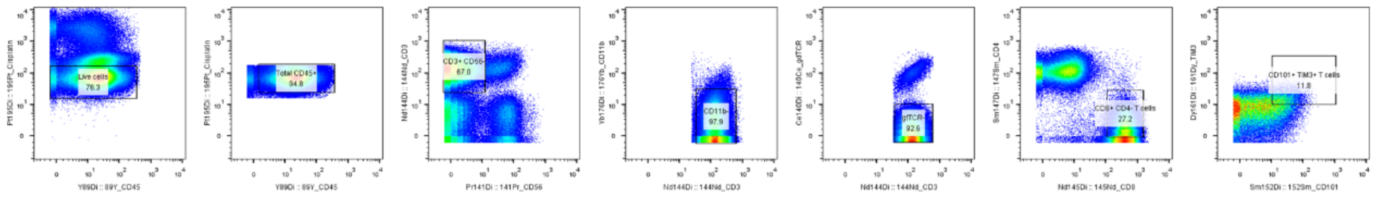

J0014A (R)

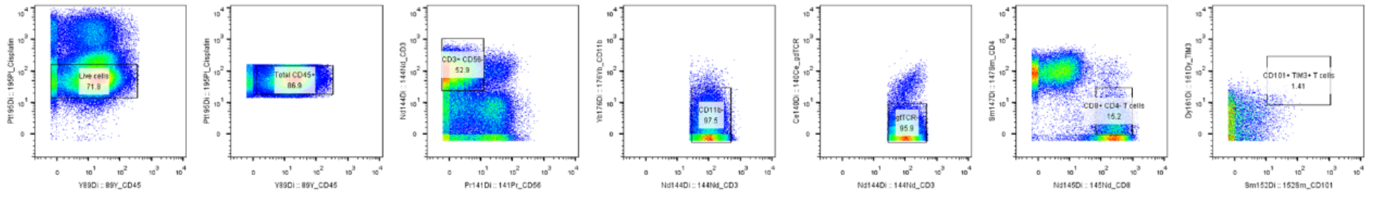

The manual gating strategy was shown below and the cluster CD8^{+} CD101^{hi} TIM3^{+} T cells can be verified by manual gating and there is significant difference between non-responders (n=14) and responders (n=11).
