# Supplementary material for: Longitudinal high-dimensional analysis identifies immune features associating with response to anti-PD-1 immunotherapy
Source: Nat Commun. 2023 Aug 22;14:5115. doi: 10.1038/s41467-023-40631-0 (PMC10444872; doi:10.1038/s41467-023-40631-0)
Supplement: Supplementary file 3 — Reporting Summary [file 41467_2023_40631_MOESM3_ESM.pdf]

nature portfolio

Corresponding author(s): Elaine Lai-Han Leung

Last updated by author(s): Jun 29, 2023

Reporting Summary

Nature Portfolio wishes to improve the reproducibility of the work that we publish. This form provides structure for consistency and transparency in reporting. For further information on Nature Portfolio policies, see our Editorial Policies and the Editorial Policy Checklist.

Statistics

For all statistical analyses, confirm that the following items are present in the figure legend, table legend, main text, or Methods section.

n/a	Confirmed	
☐	☒	The exact sample size (*n*) for each experimental group/condition, given as a discrete number and unit of measurement
☐	☒	A statement on whether measurements were taken from distinct samples or whether the same sample was measured repeatedly
☐	☒	The statistical test(s) used AND whether they are one- or two-sided<br>*Only common tests should be described solely by name; describe more complex techniques in the Methods section.*
☒	☐	A description of all covariates tested
☐	☒	A description of any assumptions or corrections, such as tests of normality and adjustment for multiple comparisons
☐	☒	A full description of the statistical parameters including central tendency (e.g. means) or other basic estimates (e.g. regression coefficient) AND variation (e.g. standard deviation) or associated estimates of uncertainty (e.g. confidence intervals)
☐	☒	For null hypothesis testing, the test statistic (e.g. *F*, *t*, *r*) with confidence intervals, effect sizes, degrees of freedom and *P* value noted<br>*Give P values as exact values whenever suitable.*
☒	☐	For Bayesian analysis, information on the choice of priors and Markov chain Monte Carlo settings
☒	☐	For hierarchical and complex designs, identification of the appropriate level for tests and full reporting of outcomes
☐	☒	Estimates of effect sizes (e.g. Cohen's *d*, Pearson's *r*), indicating how they were calculated

Our web collection on statistics for biologists contains articles on many of the points above.

Software and code

Policy information about availability of computer code

Data collection	No software was used
Data analysis	R3.6.1, GraphPad Prism 7.0, FlowJo V10

For manuscripts utilizing custom algorithms or software that are central to the research but not yet described in published literature, software must be made available to editors and reviewers. We strongly encourage code deposition in a community repository (e.g. GitHub). See the Nature Portfolio guidelines for submitting code & software for further information.

Data

Policy information about availability of data

All manuscripts must include a data availability statement. This statement should provide the following information, where applicable:

- Accession codes, unique identifiers, or web links for publicly available datasets
- A description of any restrictions on data availability
- For clinical datasets or third party data, please ensure that the statement adheres to our policy

All data generated or analyzed during this study and the dataset corresponding to the main figures is accessible in this published article (and the supplementary source, under 'Figure source'). The raw fcs files for CyTOF have been deposited in Harvard Dataverse (https://dataverse.harvard.edu/) under the Persistent Identifier: doi:10.7910/DVN/I9TGTE (https://doi.org/10.7910/DVN/I9TGTE).

Human research participants

Policy information about studies involving human research participants and Sex and Gender in Research.

Reporting on sex and gender	Our research has carefully used the term sex (biological attribute) and there was no gender bias in the conduct of investigation. Disaggregated information of sex of participants has been provided in the Patients section and Supplementary Table 1.
Population characteristics	See Supplementary Table S1. Additional information can be provided as needed.<br>Total 25 NSCLC patients with treatment of pembrolizumab were collected, among whom 19 (76%) people were male (sex) and 6 (24%) were female (sex). The mean age ± SD was 63.5 ± 12.2. As for their histology, there were 21 adenocarcinoma and 4 squamous carcinoma. Among 25 patients, 24 people were at tumor stage IV and 1 person at tumor stage IIIB. PD-L1 IHC level: >49%: 13 (52%), 1-49%: 3 (12%), <1%: 8 (32%), N/A 1 (4%).<br>Total 16 healthy donors were collected. The mean age ± SD was 55.69 ± 1.024 (min = 46, max = 62) and all of they are male potentially creating a bias for males..
Recruitment	We followed the recruitment criteria of our study protocol:<br>1. Age 18 to 80 years.<br>2. pathologically confirmed advanced non-small cell lung cancer with measurable lesions.<br>3. Receiving immunotherapy monotherapy or combined chemotherapy as first-line treatment.<br>4. ECOG PS score: 0-2.<br>5. They did not receive other systemic immunotherapy in the 4 weeks prior to enrollment.<br>All patients were recruited as described in the at Macau Kiang Wu Hospital voluntarily and they signed informed consent.<br>There is no potential self-selection bias.<br>Healthy donors were self-recruited for whole blood donations potentially creating a bias for males.
Ethics oversight	All procedures performed in studies involving human participants were in accordance with the ethical standards of the institutional and/or national research committee (Macau Kiang Wu Hospital, 2018/007).<br>informed written consent was obtained from patients included in the study.<br>A waiver of consent information for healthy person was obtained from Ethics committee of Kiang Wu Hospital.

Note that full information on the approval of the study protocol must also be provided in the manuscript.

Field-specific reporting

Please select the one below that is the best fit for your research. If you are not sure, read the appropriate sections before making your selection.

☒ Life sciences ☐ Behavioural & social sciences ☐ Ecological, evolutionary & environmental sciences

For a reference copy of the document with all sections, see nature.com/documents/nr-reporting-summary-flat.pdf

Life sciences study design

All studies must disclose on these points even when the disclosure is negative.

Sample size	Sample size were were chosen according to sample availability. 25 patients with non-small cell lung cancer at Macau Kiang Wu Hospital received anti-PD1 treatment over the last 30 months. Among all samples analyzed we include data from 102 PBMC and 121 serum samples.
Data exclusions	Poor quality samples (low cells number, high dead cells number) were not included in the study.
Replication	All attempts at replication were successful by 102 PBMC samples and 121 serum samples.
Randomization	This is phase II single-arm, non-randomization and open study. All patients received same treatment regimens, no investigational treatment was used.<br>Thus, the data presented did not require the use of randomization.
Blinding	25 lung cancer patients were collected who received anti-PD-1 treatment and immunophenotyping, molecular analysis and functional characterization of immune populations were performed.<br>All patients received the same treatment according to the treatment protocol, and no other investigative treatments were administered in addition to the anti-PD-1 treatment.<br>As our aim was to investigate the predictive and resistant biomarkers associated with anti-PD-1 treatment, blinding was not necessary for the data presented.

Reporting for specific materials, systems and methods

We require information from authors about some types of materials, experimental systems and methods used in many studies. Here, indicate whether each material, system or method listed is relevant to your study. If you are not sure if a list item applies to your research, read the appropriate section before selecting a response.

Materials & experimental systems

n/a	Involved in the study
☐	☒ Antibodies
☒	☐ Eukaryotic cell lines
☒	☐ Palaeontology and archaeology
☒	☐ Animals and other organisms
☐	☒ Clinical data
☒	☐ Dual use research of concern

Methods

n/a	Involved in the study
☒	☐ ChIP-seq
☒	☐ Flow cytometry
☒	☐ MRI-based neuroimaging

Antibodies

Antibodies used

Antibody Clone Provider Cat# Dilution (x-fold)
CD45 HI30 Fluidigm 3089003B 100
CD14 TUK4 Invitrogen Q10064 100
CD15 HI98 Biolegend bl301902 100
Anti-PE PE001 Biolegend bl408102 100
TCRgt-PE 5A6.E9 Invitrogen MHGD04 25
CD56 NCAM16.2 BD bd559043 100
CD19 HIB-19 Biolegend bl302202 100
CD27 LG.7F9 Biolegend inv14-0271-85 100
CD3 UCHT1 Biolegend bl300414 100
CD8 SK1 Biolegend bl344701 100
HLA-DR L243 Biolegend bl307602 100
CD4 SK3 Biolegend bl344602 100
CD45RO UCHL1 Biolegend bl304202 50
ICOS C398.4A Biolegend bl313502 50
Granzyme B CLB-GB11 Abcam ab103159 800
CD69 FN50 Biolegend bl310902 100
CD101 BB27 eBioscience 14101982 100
KLRG1 13F2F12 eBioscience ebio16-9488-85 100
CXCR5 RF8B2 BD bd552032 100
CD33 WM53 Biolegend 303402 100
Tbet 4B10 Biolegend bl644802 100
CXCR3 MAB160 R&D MAB160-100 100
FOXP3-biot PCH101 eBioscience 13-4776-82 100
PDL1 29E.2A3 Biolegend bl329710 25
PD-1 eBioJ105 eBioscience eBio14-2799-80 50
TIM-3 344823 R&D MAB2365 100
CD95 DX2 Biolegend 305602 100
CD127 AO19D5 Biolegend bl351302 100
IgG4 Fc-UNLB Southern Biotech 9200-01 100
Ki67 B56 BD bd556003 100
Ox40 443318 R&D MAB3388 50
GATA3 TWAJ eBioscience ebio14-9966-82 100
CCR7 150503 R&D MAB197 50
CD25 M-A251 Biolegend bl356102 100
CTLA4 14D3 eBioscience ebio14-1529-82 25
CD28 CD28.2 Biolegend bl302914 50
CD38 HIT2 Biolegend bl303502 100
CD39 A1 Biolegend bl328202 100
Eomes WD1928 eBioscience inv14-4877-82 50
CD11c B-Ly6 BD bd555390 100
CD11b ICRF44 Biolegend bl301312 100
CD16 3g8 Fluidigm 3209002B 100

Validation

Validation of all Antibodies.
All antibodies were titrated and tested by assessing the relative marker expression intensity on relevant immune cell subsets in PBMCs from healthy individuals.
Antibody validation of the manufacturers were available for all antibodies including positive and negative staining controls.
(Additional info could be found under the Cat# on the respective providers page)

Clinical data

Policy information about clinical studies

All manuscripts should comply with the ICMJE guidelines for publication of clinical research and a completed CONSORT checklist must be included with all submissions.

Clinical trial registration	Macau Kiang Wu Hospital (2018-007)
Study protocol	Macau Kiang Wu Hospital Ethic committee
Data collection	All data of patients were collected from Macau Kiang Wu Hospital from 2018 to 2020.
Outcomes	Clinical pathological data, routine CT scans, and immunohistochemistry (IHC) data were recorded. Immunological profiles and patient response correlation analysis were applied.
